# Provider-mother interactions are associated with birth outcome misclassifications in household surveys: A case-control study in Guinea-Bissau

**DOI:** 10.7189/jogh.13.04086

**Published:** 2023-08-18

**Authors:** Sabine M Damerow, Diana Yeung, Justiniano SD Martins, Ishaan Pathak, Yue Chu, Li Liu, Ane B Fisker

**Affiliations:** 1 Bandim Health Project, INDEPTH Network, Bissau, Guinea-Bissau; 2 Bandim Health Project, Research Unit OPEN, Department of Clinical Research, University of Southern Denmark, Odense, Denmark; 3 Institute for International Programs, Department of International Health, Johns Hopkins Bloomberg School of Public Health, Baltimore, Maryland, USA; 4 Department of Sociology, The Ohio State University, Columbus, Ohio, USA; 5 Institute for Population Research, The Ohio State University, Columbus, Ohio, USA; 6 Population, Family, and Reproductive Health, Johns Hopkins University Bloomberg School of Public Health, Baltimore, Maryland, USA

## Abstract

**Background:**

Approximately 4.4 million children die peripartum annually, primarily in low- and middle-income countries. Accurate mortality tracking is essential to prioritising prevention efforts but is undermined by misclassification between stillbirths (SBs) and early neonatal deaths (ENNDs) in household surveys, which serve as key data sources. We explored and quantified associations between peripartum provider-mother interactions and misclassification of SBs and ENNDs in Guinea-Bissau.

**Methods:**

Using a case-control design, we followed up on women who had reported a SB or ENND in a retrospective household survey nested in the Bandim Health Project’s Health and Demographic Surveillance Systems (HDSS). Using prospective HDSS registration as the reference standard, we linked the survey-reported deaths to the corresponding HDSS records and cross-tabulated SB/ENND classification to identify cases (discordant classification between survey and HDSS) and controls (concordant classification). We further interviewed cases and controls on peripartum provider-mother interactions and analysed data using descriptive statistics and logistic regressions.

**Results:**

We interviewed 278 women (cases: 63 (23%); controls: 215 (77%)). Most cases were SBs misclassified as ENNDs (n/N = 49/63 (78%)). Three-fourths of the interviewed women reported having received no updates on the progress of labour and baby’s health intrapartum, and less than one-fourth inquired about this information. In comparison with births where women did inquire for information, misclassification was less likely when women did not inquire and recalled no doubts about progress of labour (odds ratio (OR) = 0.51; 95% confidence interval (CI) = 0.28-0.91), or baby’s health (OR = 0.54; 95% CI = 0.30-0.97). Most women reported that service providers’ death notifications lasted <5 minutes (cases: 23/27 (85%); controls: 61/71 (86%)), and most often encompassed neither events leading to the death (cases: 19/27 (70%); controls: 55/71 (77%)) nor causes of death (cases: 20/27 (74%); controls: 54/71 (76%)). Misclassification was more likely if communication lasted <1 compared to 1-4 minutes (OR = 1.83; 95% CI = 1.10-3.06) and if a formal service provider had informed the mother of the death compared to a family member (OR = 1.57; 95% CI = 1.04-2.36).

**Conclusions:**

Peripartum provider-mother interactions are limited in Guinea-Bissau and associated with birth outcome misclassifications in retrospective household surveys. In our study population, misclassification led to overestimated neonatal mortality.

An estimated two million children are stillborn and 2.4 million die during their first month of life globally each year [1], 97% of them in low- and middle-income countries (LMICs) [2]. Regionally, sub-Saharan Africa and South Asia bear the greatest burden, with regional estimates exceeding 1.5-times the Every Newborn Action Plan target of no more than 12 stillbirths per 1000 births [3,4] and 1.9-times the Sustainable Development Goal target of no more than 12 neonatal deaths per 1000 live births [1,5]. Concerted action is needed to lower perinatal mortality in these settings.

Accurate and timely mortality tracking is essential for prioritising mortality prevention efforts and monitoring progress towards the global mortality targets [6-9]. However, while civil registration and vital statistics (CRVS) commonly provide a reliable data source for mortality tracking in high-income settings [10], births and deaths often remain unregistered in LMICs, resulting in inadequate CRVS in many high-burden countries [6,11-13]. In these settings, perinatal mortality tracking remains reliant on periodically conducted large-scale household surveys such as the Demographic Health Surveys (DHS) or Multiple Indicator Cluster Surveys [14,15].

Such surveys have most commonly used the full birth history (FBH) approach to capture mortality. In FBHs, women are interviewed on their history of live births, child survival status, and child age at death, thereby omitting adverse non-live pregnancy outcomes including stillbirths [14]. To capture non-live pregnancy outcomes, FBH surveys can be complemented by probes on pregnancy losses (FBH^+^) [16]. More recently, the full pregnancy history (FPH) approach has gained increased attention. Here, women are interviewed on their history of pregnancies, child survival status, and child age at death, thereby directly capturing both live and non-live pregnancy outcomes, including stillbirths [14,16]. Since 2020 (DHS-VIII), the FPH approach has been adopted in the DHS standard questionnaire [14,17].

There are several data quality challenges associated with survey-derived stillbirth and neonatal mortality estimates [14,18,19], one being misclassification between pregnancy outcomes – for example, stillbirths being incorrectly classified as neonatal deaths, or vice versa [18]. In a validation study comparing FBH^+^ survey data with that from a prospectively followed health and demographic surveillance system (HDSS) cohort in urban Guinea-Bissau, the FBH^+^ data overestimated neonatal mortality by 5-20% [18]. Most errors resulted from a misclassification between early neonatal deaths and stillbirths, contributing to 43% of neonatal deaths that were reported as such in HDSS, but not in FBH^+^ data, and 63% of deaths reported in FBH^+^, but not in HDSS data [18]. Similarly, a study validating FBH-reported neonatal deaths through verbal and social autopsies in Malawi found that 21% of all FBH-reported neonatal deaths were likely misclassified stillbirths [20].

Accurate classification of stillbirths and neonatal deaths based on household surveys presupposes maternal knowledge of the child’s vital status at birth. Several determinants of maternal misclassification have been discussed, including compromised recall and numeracy [18], social desirability, stigma associated with specific birth outcomes, and linguistic ambiguity between outcomes [21]. However, provider-mother interactions around the time of birth have received little attention, despite evidence reflecting various provider practices which may impact accurate maternal knowledge of the child’s vital status at birth, including avoiding or delaying informing the mother about the child death [22], avoiding showing her the deceased child [22-24], spending little time on providing information about the death [25], and more generally, poor communication [26-29]. Therefore, we aimed to explore and quantify associations between peripartum provider-mother interactions and maternal misclassification of stillbirths and early neonatal deaths in Guinea-Bissau.

## METHODS

### Study design

We applied an unmatched case-control design, defining cases and controls based on discordant (cases) and concordant (controls) maternal reporting of a child death as stillbirth or early neonatal death (i.e. death during the first week of life) between two data sources: a retrospective population survey mimicking DHS data collection using standard FBH^+^ or FPH modules (EN-INDEPTH study) conducted at the Bandim Health Project (BHP) in 2017-2018 [16], and prospective mortality surveillance of BHP’s routine HDSS [30,31]. To identify cases and controls, we linked records from both data sources at the individual level (Methods S1 in the **Online Supplementary Document**). HDSS classification of stillbirths and early neonatal deaths served as the reference standard. We sought to include all linked cases and controls. When planning the study, we anticipated to include 103 cases and 348 controls, and estimated that this would give us 80% power to identify significant risk factors if the underlying distribution of a binary risk factor differed by ~ 15 percentage points and the factor was present for ~ 50%.

### Study population

We recruited participants in 2021-2022 from respondents of the EN-INDEPTH study, which collected retrospective self-reported information on stillbirths and early neonatal deaths among women in BHP’s urban and rural HDSS with a registered birth outcome during the last five years [16,32]. BHP’s HDSS prospectively monitors maternal and child health outcomes in an open cohort of over 45 000 women and 43 000 children in urban and rural Guinea-Bissau through regular household visits [30,31]. If a cohort member is absent at a household visit, related persons (usually female household members) are asked to provide information on behalf of the woman. The women eligible for study participation were EN-INDEPTH respondents who reported at least one birth resulting in stillbirth or early neonatal death in the five years prior to the EN-INDEPTH survey which was also registered in BHP’s HDSS (n = 391).

### Data collection

We invited eligible women residing in the HDSS area to participate in a household interview (case-control interview). They were initially contacted by the HDSS staff routinely collecting household information and asked if they were willing to be visited by a colleague collecting more information on women’s experiences of losing a child. Provided acceptance, one of two specially trained female HDSS field workers visited the women for the case-control interviews. Following a consent process (Methods S2. in the **Online Supplementary Document**), the HDSS field worker collected information via a tablet-based structured questionnaire with items on background characteristics and peripartum provider-mother interactions (Questionnaire S1 in the **Online Supplementary Document**). Background characteristics included maternal information (e.g. prior adverse birth outcomes), antenatal care (ANC) information (e.g. number of ANC consultations obtained), and birth and perinatal death information (e.g. time and place of delivery). Peripartum provider-mother interactions focussed on two time points – labour and delivery (communication on progress of labour and baby’s/mother’s health, maternal worries, and postpartum interactions with the baby), and the notification about the child death (informant, context, language, comprehensibility, timing, and informant’s behaviour). We also inquired about the communicated and self-observed child’s vital status at birth to triangulate between data sources. We developed the questions in English and translated them to Portuguese, the written language of Guinea-Bissau. Research team members supervised the data collection which was implemented in Guinea-Bissau Creole. Interviews were conducted at a location chosen by the respondent, usually the respondent’s residence. Interviewers were blinded to case/control classifications. Interviews lasted for approximately 30 minutes.

We also extracted HDSS-recorded information on birth outcome, informant, recall length, and residency, and EN-INDEPTH-recorded information on birth outcome, recall length, survey module, maternal age and education, household assets, and ethnicity (Table S1-S2 in the **Online Supplementary Document**). We also extracted information on the facility-recorded vital status at birth for all women who gave birth at Guinea-Bissau’s National Hospital Simão Mendes (HNSM) [30] to further triangulate self- and facility-reported information (Table S1 in the **Online Supplementary Document**).

### Data analyses

We compared stillbirth and early neonatal death classifications across data sources by cross tabulating the outcomes recorded in EN-INDEPTH, HDSS, HNSM, and case-control interview data. Using bivariate analyses, we described background characteristics across cases and controls and compared distributions between cases and controls using χ^2^ tests. We examined bivariate associations between peripartum provider-mother interactions (Table S3 in the **Online Supplementary Document**) and misclassification using logistic regression and conducted sub-analyses adjusting the bivariate associations for background characteristics selected based on field observations of potential confounders (residency, maternal education, parity/prior adverse birth outcomes, recall periods, facility birth, C-section, intrapartum complications, place of death, proxy reporting) (Table S2 in the **Online Supplementary Document**). Due to the limited sample size, we ran the adjusted analyses for one background factor at a time. We set the statistical significance threshold for all analyses at a significance level of *P* < 0.05. To account for possible cluster effects stemming from the selection of HDSS participants based on their residency in a defined geographical area, we used Stata survey commands (SVY) in all statistical tests (Methods S4 in the **Online Supplementary Document**). All analyses were complete case, so we excluded observations with “Unknown” responses from the analyses but chose to display them for completeness. We also assessed associations between provider-mother interactions and misclassification in restricted samples: only births attended by a skilled or traditional attendant, and only facility births. We performed all analyses in Stata 17.0 (StataCorp, College Station TX, USA).

## RESULTS

**Figure 1:**
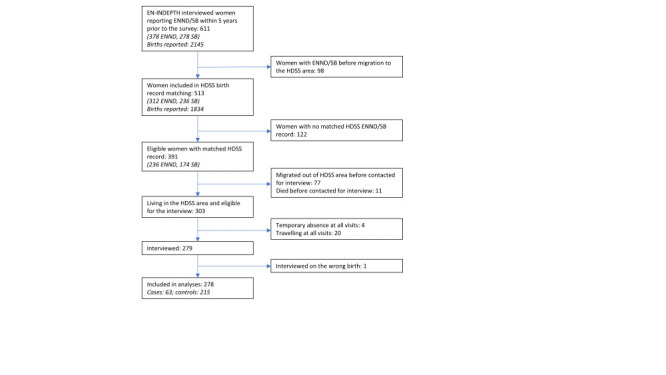
Inclusion of study participants. EN-INDEPTH – EN-INDEPTH study conducted at the Bandim Health Project in 2017-18 [16], ENND – early neonatal death, HDSS – health and demographic surveillance system of the Bandim Health Project, SB – stillbirth.

Among the 391 eligible women with matched EN-INDEPTH and HDSS records, 77 migrated out of the HDSS study area, 11 died before being contacted for the case-control interview, and 24 were absent at all household visits. Among the 279 women interviewed, we excluded one record since the woman was interviewed on a wrong birth. Our final sample included 278 women (cases: 63; controls: 215) (**Figure 1**). There were no differences in the background characteristics between interviewed and potentially eligible but not interviewed women, except that the latter were slightly younger (Table S4 in the **Online Supplementary Document**).

**Figure 2:**
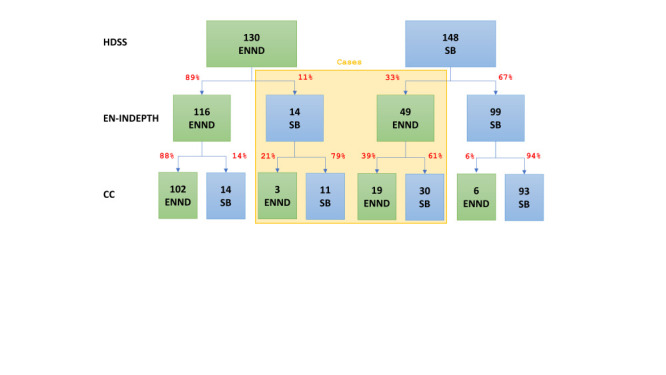
Classification of stillbirths and early neonatal deaths across data sources. CC – case-control interview, EN-INDEPTH – EN-INDEPTH study conducted in 2017-18 [16], ENND – early neonatal death, HDSS – health and demographic surveillance system, SB – stillbirth.

In the HDSS data, 130 deaths were classified as early neonatal deaths and 148 as stillbirths. While 89% of the HDSS-classified early neonatal deaths were concordantly classified in EN-INDEPTH data (n/N = 116/130), this was the case for only 67% of the HDSS-classified stillbirths (n/N = 99/148) (**Figure 2** and Table S5 in the **Online Supplementary Document**). Discrepancies between HDSS and EN-INDEPTH classifications were similar across the two different EN-INDEPTH survey modules (FBH^+^ and FPH) (Table S5 in the **Online Supplementary Document**). Classifications were less discordant between HDSS data and the case-control interviews; 83% of the HDSS-classified stillbirths and 81% of early neonatal deaths (n/N = 105/130) were concordantly classified in the case-control interviews (**Figure 2** and Table S5 in the **Online Supplementary Document**). HDSS classification was most concordant with HNSM classification (stillbirths: 54/59 (92%); early neonatal deaths: 31/36 (86%)) (Table S5 and Figure S1 in the **Online Supplementary Document**).

Most maternal characteristics were similarly distributed across cases and controls. Two-thirds of the interviewed women lived in the urban study area (n = 193 (69%)), and close to half of the women were aged 20-29 years at the time of the EN-INDEPTH interview (n = 132 (47%)). The overall level of educational attainment was low, with 35% of the women never having attended school (n = 96) and 28% having attended only primary school (n = 79). Three-quarter of the women had at least one pregnancy prior to the one interviewed about (n = 207 (74%)), and 29% of those women had experienced a previous perinatal death (n = 60). Based on wealth quintiles, women from the urban study area appeared poorer than those from the rural areas. Fula/Mandinga were the biggest ethnic group (n = 82 (29%)), followed by Pepel (n = 66 (24%)) (**Table 1**).

**Table 1:** Maternal characteristics*

	Cases	Controls	Total	*P*-value†
**Overall**	63 (23)	215 (77)	278 (100)	
**Residency**				0.324
Rural	22 (35)	63 (29)	85 (31)	
Urban	41 (65)	152 (71)	193 (69)	
**Maternal age**				0.616
<20 y	8 (13)	31 (14)	39 (14)	
20-29 y	28 (44)	104 (48)	132 (47)	
30-39 y	25 (40)	71 (33)	96 (35)	
>40 y	2 (3)	9 (4)	11 (4)	
**Highest level of school attained**				0.485
No schooling	24 (38)	72 (33)	96 (35)	
Primary	19 (30)	60 (28)	79 (28)	
Secondary or higher	20 (32)	83 (39)	103 (37)	
**Parity**				0.064
Primigravida	19 (30)	52 (24)	71 (26)	
Multipara (1+ prior births)	44 (70)	163 (76)	207 (74)	
**Prior adverse birth outcome‡**				0.072
Yes, previous SB/ENND	16 (36)	44 (27)	60 (29)	
No	28 (64	118 (72)	146 (71)	
Unknown§	0 (0)§	1 (1)§	1 (0)§	
**Wealth quintile urban‖**				0.210¶
Poorest	9 (22)	35 (23)	44 (23)	
2	10 (24)	54 (36)	64 (33)	
3	11 (27)	42 (28)	53 (27)	
4	11 (27)	21 (14)	32 (17)	
Wealth quintile rural**				0.578
4	7 (32)	16 (25)	23 (27)	
Richest	15 (68)	47 (75)	62 (73)	
**Ethnicity**				0.522
Balante	5 (8)	26 (12)	31 (11)	
Fula/Mandinga	19 (30)	63 (29)	82 (29)	
Mancanha/Manjaco	9 (14)	36 (17)	45 (16)	
Pepel	18 (29)	48 (22)	66 (24)	
Other/mixed	12 (19)	42 (20)	54 (19)	

ENND – early neonatal death, SB – stillbirth, y – year

*Values presented as n (%) unless otherwise specified.

†Based on χ^2^ adjusted for possible cluster effects associated with respondents' residency.

‡Among all women with ≥1 previous pregnancy (n = 207).

§Shown for completeness but excluded from statistical comparison.

‖Among all women with urban residence (n = 193).

¶Not adjusted for residency since all urban residents belong to one single stratum.

**Among all women with rural residence (n = 85).

Most women gave birth at a health facility (n = 232 (83%)) and had been assisted by a skilled birth attendant (n = 225 (81%)). Among the women giving birth in a health facility, one in five had been referred (n = 49 (21%)). Most women had given birth vaginally (n = 221 (79%)), 15% had a C-section (n = 43). One in five women reported labour of >18 hours (n = 59 (21%)), and more than half reported intrapartum complications (breech or transverse position, umbilical cord prolapse, nuchal cord, excessive bleeding) (any: n = 115 (41%); >1: n = 30 (11%)). Nine per cent of the women had a multiple gestation (n = 24). Most perinatal deaths occurred at the health facility of delivery (n = 184 (66%)), 21% at home (n = 57), and 9% at another health facility (n = 25) (**Table 2**). Most women reported having had some risk factors during their pregnancy (any: n = 237 (85%)) but had not been informed about dangers to the baby’s health by a health professional before delivery (n = 193 (83%)). The provision of information on danger signs differed statistically significantly between cases and controls (*P* = 0.015) with more controls than cases having been informed about serious dangers to the baby’s health (controls: n = 21 (12%); cases: n = 3 (5%)) (Table S6 in the **Online Supplementary Document**). None of the other birth, perinatal death, and ANC characteristics differed statistically significantly between cases and controls (**Table 2**) (Table S6 in the **Online Supplementary Document**).

**Table 2:** Place and characteristics of birth and death*

	Cases	Controls	Total	*P*-value†
**Overall**	63 (23)	215 (77)	278 (100)	
**Facility birth**				0.138
Yes	56 (89)	176 (82)	232 (83)	
No	7 (11)	39 (18)	46 (17)	
**Birth attendant**				0.618
SBA	53 (84)	172 (80)	225 (81)	
TBA	4 (6)	14 (7)	18 (6)	
Other	6 (10)	29 (13)	35 (13)	
**Referred to HF‡**				0.339
Yes	14 (25)	35 (20)	49 (21)	
No	41 (73)	141 (80)	182 (78)	
Unknown§	1 (2)§	0 (0)§	1 (0)§	
**Type of birth**				0.104
Vaginally	50 (79)	171 (80)	221 (79)	
Instrumental vaginally	1 (2)	11 (5)	12 (4)	
C-Section	10 (16)	33 (15)	43 (15)	
Unknown§	2 (3)§	0 (0)§	2 (1)§	
**Duration of labour and birth**				0.417
<6h	23 (37)	77 (36)	100 (36)	
6-11h	12 (19)	51 (24)	63 (23)	
12-18h	13 (21)	34 (16)	47 (17)	
>18h	12 (19)	47 (22)	59 (21)	
Unknown§	3 (5)§	6 (3)§	9 (3)§	
**Time of birth**				0.073
Daytime (8 AM to 4 PM)	19 (30)	73 (34)	92 (33)	
Evening (4 PM to 12 AM)	17 (27)	71 (33)	88 (32)	
Late night (12 AM to 8 AM)	24 (38)	63 (29)	87 (31)	
Unknown§	3 (5)§	8 (4)§	11 (4)§	
**Multiple gestation**				0.370
Single	59 (94)	195 (91)	254 (91)	
Multiple	4 (6)	20 (9)	24 (9)	
**Intrapartum complications**				0.621
None reported	29 (46)	104 (48)	133 (48)	
1 reported	28 (44)	87 (40)	115 (41)	
>1 reported	6 (10)	24 (11)	30 (11)	
**Baby moved before birth**				0.658
Yes	53 (84)	183 (85)	236 (85)	
No	9 (14)	27 (13)	36 (13)	
Unknown§	1 (2)§	5 (2)§	6 (2)§	
**Mother noticed birth injury‖**				0.601
Yes	2 (5)	10 (6)	12 (6)	
No	36 (88)	134 (86)	170 (86)	
Unknown§	3 (7)§	12 (8)§	15 (8)§	
**Place of baby's death**				0.128
HF of delivery	49 (78)	135 (63)	184 (66)	
Other HF	1 (2)	24 (11)	25 (9)	
Home	11 (17)	46 (21)	57 (21)	
Other	1 (2)	9 (4)	10 (4)	
Unknown§	1 (2)§	1 (0)§	2 (1)§	

HF – health facility, SBA – skilled birth attendant, TBA – traditional birth attendant

*Values presented as n (%) unless otherwise specified.

†Based on χ^2^ adjusted for possible cluster effects associated with respondents' residency.

‡Among all women who gave birth at a health facility (n = 232).

§Shown for completeness but excluded from statistical comparison.

‖Among all women who had seen the baby postpartum (n = 198), one missing value.

Recall lengths varied substantially between data sources. While HDSS interviews were conducted at a median of 0.6 months after birth (interquartile range (IQR) = 0.1-1.2), EN-INDEPTH interviews were conducted at a median of 26 months (IQR = 9-43) and case-control interviews at a median of 74 months (IQR = 59-92). While the distribution of women interviewed above the respective median recall was similar across cases and controls for EN-INDEPTH interviews and case-control interviews, cases tended to have a longer recall length in HDSS interviews (*P* = 0.053). Proxy reporting of HDSS data was more common among cases than controls (*P* = 0.044). The distribution of women who had been interviewed using FBH^+^ and FPH surveys in the EN-INDEPTH interviews was even and similarly distributed across cases and controls (Table S7 in the **Online Supplementary Document**).

For intrapartum provider-mother interactions, three-fourths of both cases and controls reported that they had not received any updates on the progress of labour and the baby’s and mother’s health. Although a slightly lower proportion of both cases and controls reported that they had been worried about their baby's health, less than one in four cases reported having inquired information about the progress of labour and the baby’s health, and the proportion was lower among controls (progress of labour – cases: 15 (24%); controls: 31 (14%); baby’s health – cases: 13 (21%); controls: 31 (14%)). In comparison with deaths where the mother inquired information about the progress of labour and the baby’s health, misclassification was less likely when the mother recalled no doubts (progress of labour: OR = 0.51; 95% CI = 0.28-0.91, and baby’s health: OR = 0.54; 95% CI = 0.30-0.97). Most women saw, heard, or felt their baby postpartum (cases: 41 (65%); controls: 157 (73%)) and misclassification tended to be more likely when the mother did not see, hear, or feel the baby (OR = 1.48; 95% CI = 0.85-2.57) (**Table 3**).

**Table 3:** Intrapartum provider-mother interactions*

	Cases	Controls	OR (95% CI)†
**Overall**	63 (23)	215 (77)	
**Updates obtained on baby’s/women’s health and progress of labour**			
Any	11 (17)	36 (17)	ref.
More comprehensive	5 (8)	16 (7)	1.02 (0.38-2.78)
None	47 (75)	163 (76)	0.94 (0.54-1.64)
**Mother was worried about baby's health**			
No	18 (29)	64 (30)	ref.
Yes, little/average	24 (38)	81 (38)	1.05 (0.63-1.77)
Yes, very/extremely	21 (33)	70 (33)	1.07 (0.62-1.82)
**Mother inquired progress of labour**			
Yes, asked	15 (24)	31 (14)	ref.
No, unable	31 (49)	115 (53)	0.56 (0.29-1.06)
No, no doubts recalled	17 (27)	69 (32)	0.51 (0.28-0.91)
**Mother inquired baby's health during labour**			
Yes, asked	13 (21)	31 (14)	ref.
No, unable	35 (56)	118 (55)	0.71 (0.39-1.28)
No, no doubts recalled	15 (24)	66 (31)	0.54 (0.30-0.97)
**Mother saw, heard or felt baby postpartum**			
Yes	41 (65)	157 (73)	ref.
No	22 (35)	57 (27)	1.48 (0.85-2.57)
Unknown‡	0 (0)‡	1 (0)‡	

OR – odds ratio, CI – confidence interval, ref. – reference

*Values presented as n (%) unless otherwise specified.

†Adjusted for possible cluster effects associated with respondents' residency.

‡Shown for completeness but excluded from the calculation of ORs.

Regarding provider-mother interactions during the communication of the baby’s death, while slightly more than two-thirds of both cases and controls were directly informed (cases: 45 (71%); controls: 149 (69%)), 17% of cases (n = 11) and 23% of controls (n = 50) learned about the death indirectly, e.g. by seeing the dead child or overhearing a conversation between service providers and family members. Misclassification tended to be less likely when the woman was not directly informed (OR = 0.73; 95% CI = 0.51-1.03). Among women who were directly informed, half were informed by a formal service provider (cases: 24 (53%); controls: 67 (45%)), and misclassification was more likely if a formal service provider had informed in comparison with a family member (OR = 1.57; 95% CI = 1.04-2.36). Half of the women reported that family was present during the death notification. Misclassification was more likely amongst women who had met the informing provider once before compared with women who did not know the provider (OR = 4.58; 95% CI = 1.51-13.91), but not among women who had met the provider more often. Misclassification was less likely among mothers who learned about the death at the health facility outside the delivery room and maternity ward compared with in the delivery room (OR = 0.18; 95% CI = 0.05-0.58) (**Table 4**); the place of communication and how long after delivery the communication took place were correlated (data not shown).

**Table 4:** Provider-mother interactions during communication of baby’s death*

	**Cases**	**Controls**	**OR (95% CI)†**
**Overall**	63 (23)	215 (77)	
**Source of information**			
Death was directly communicated to the mother			
*Yes*	45 (71)	149 (69)	ref.
*No*	11 (17)	50 (23)	0.73 (0.51-1.03)
*Unknown‡*	7 (11)‡	16 (7)‡	
First person who communicated the death to the mother§			
*Family member/partner*	16 (36)	70 (47)	ref.
*Formal service provider*	24 (53)	67 (45)	1.57 (1.04-2.36)
*Informal service provider*	3 (7)	7 (5)	1.88 (0.36-9.77)
*Other*	2 (4)	5 (3)	1.75 (0.39-7.91)
Mother's familiarity with the provider‖			
*None*	16 (59)	55 (76)	ref.
*1 previous encounter*	8 (30)	6 (8)	4.58 (1.51-13.91)
*≥2 previous encounters*	3 (11)	11 (15)	0.94 (0.20-4.38)
**Context**			
Mother's location when learning about death			
*Delivery room*	14 (22)	41 (19)	ref.
*Maternity ward*	35 (56)	86 (40)	1.19 (0.67-2.11)
*At home*	9 (14)	44 (20)	0.60 (0.25-1.43)
*Another place in the HF*	2 (3)	33 (15)	0.18 (0.05-0.58)
*Another place outside the HF*	1 (2)	4 (2)	0.73 (0.09-6.23)
*Unknown‡*	2 (3)‡	7 (3)‡	
Presence of other people			
*Only other unknown people*	20 (32)	63 (29)	ref.
*Family and other unknown people*	19 (30)	54 (25)	1.11 (0.71-1.72)
*Family only*	14 (22)	50 (23)	0.88 (0.51-1.53)
*The woman was alone*	7 (11)	34 (16)	0.65 (0.35-1.19)
*Unknown‡*	3 (5)‡	14 (7)‡	
**Language, comprehensibility, and completeness**			
Language spoken‖			
*Guinea-Bissau Creole*	24 (89)	69 (96)	ref.
*Portuguese*	1 (4)	0 (0)	NA
*Other*	2 (7)	3 (4)	1.92 (0.28-13.06)
Comprehensibility of information provided¶			
*Yes, understood immediately*	23 (85)	60 (85)	ref.
*Yes, but more information needed*	1 (4)	2 (3)	1.30 (0.07-24.68)
*No, vague statement only*	3 (11)	8 (11)	0.98 (0.29-3.34)
*No, medical language*	0 (0)	1 (1)	NA
Comprehensibility of events leading to death¶			
*Yes, understood immediately*	8 (30)	14 (20)	ref.
*Yes, but more information needed*	0 (0)	1 (1)	NA
*Not mentioned*	19 (70)	55 (77)	0.60 (0.16-2.26)
*No, medical language*	0 (0)	1 (1)	NA
Comprehensibility of causes of death¶			
*Yes, understood immediately*	6 (22)	14 (20)	ref.
*Yes, but more information needed*	0 (0)	1 (1)	NA
*Not mentioned*	20 (74)	54 (76)	0.86 (0.28-2.67)
*No, medical language*	0 (0)	1 (1)	NA
*Unknown‡*	1 (4)‡	1 (1)‡	
Completeness of information¶			
*Yes, sufficient*	21 (78)	55 (77)	ref.
*No, too much*	1 (4)	1 (1)	2.62 (0.13-53.08)
*No, not enough*	4 (15)	13 (18)	0.81 (0.33-1.95)
*Unknown‡*	1 (4)‡	2 (3)‡	
Duration of information provision¶			
*<1 min*	14 (52)	28 (39)	1.83 (1.10-3.06)
*1-4 min*	9 (33)	33 (46)	ref.
*≥5 min*	2 (7)	3 (4)	2.44 (0.34-17.43)
*Unknown‡*	2 (7)‡	7 (10)‡	
**Provider behaviour**			
Mother recalls being comforted by the provider¶			
*Yes*	24 (89)	60 (85)	ref.
*No*	3 (11)	11 (15)	0.68 (0.18-2.62)
Mother recalls that the provider was dismissive¶			
*Yes*	1 (4)	4 (6)	0.64 (0.09-4.79)
*No*	26 (96)	67 (94)	ref.
Mother recalls that the provider gave no counselling¶			
*Yes*	4 (15)	10 (14)	1.06 (0.50-2.23)
*No*	23 (85)	61 (86)	ref.
Informant paid full attention to mother while informing about baby's death‖			
*Paid full attention to mother*	24 (89)	60 (83)	ref.
*Was distracted*	1 (4)	5 (7)	0.50 (0.16-1.59)
*Unknown‡*	2 (7)‡	7 (10)‡	

HF – health facility, ref. – reference, NA – not applicable, OR – odds ratio, CI – confidence interval

*Values presented as n (%) unless otherwise specified.

†Adjusted for possible cluster effects associated with respondents' residency.

‡Shown for completeness but excluded from the calculation of ORs.

§Among all women to whom the death was directly communicated (n = 194); the categories include values that were initially coded as “other” and could be reassigned based on their free-text specification: formal service provider: n = 1; informal service provider: n = 1; family member: n = 86.

‖Among all women who were initially coded as being informed by a formal/informal service provider during the interview (n = 99).

¶Among all women who were initially coded as being informed by a formal/informal service provider during the interview (n = 99), one missing value.

Most women reported that service providers’ death notifications were short and lasted <5 minutes (cases: 23/27 (85%); controls: 61/71 (86%)). Misclassification was more likely if the communication was <1 minute compared to 1-4 minutes (OR = 1.83; 95% CI = 1.10-3.06). The majority of both cases and controls reported that the service provider communicated the death in a way they understood immediately (cases: 23/27 (85%); controls: 60/71 (85%)), told everything they wanted to know (cases: 21/27 (78%); controls: 55/71 (77%)), and paid full attention to them (cases: 24/27 (89%); controls: 60/72 (83%)). Yet most women also reported that the service providers neither mentioned events leading to the death (cases: 19/27 (70%); controls: 55/71 (77%)) nor causes of death (cases: 20/27 (74%); controls: 54/71 (76%)) (**Table 4**).

Among the assessed potential confounders, parity/prior adverse birth outcome (prior loss of a child: OR = 1.53; 95% CI = 0.96-2.44 or being primigravida: OR = 1.54; 95% CI = 1.09-2.18 vs prior birth without loss), and proxy reporting (OR = 1.70; 95% CI = 1.01-2.88) were associated with higher odds of misclassification (Table S8 in the **Online Supplementary Document**). However, adjusting for these and the other potential confounders in the bivariate analyses did not alter conclusions (Figures S2-S12 in the **Online Supplementary Document**). Limiting the analyses to facility births or births with a skilled/traditional birth attendant did not alter conclusions either (data not shown).

## DISCUSSION

In this study, we found limited peripartum provider-mother interactions during births with adverse outcomes in Guinea-Bissau: three-fourths of our study participants reported not having been updated on either the progress of labour or the baby’s health during birth, and less than one-fourth had inquired about this information. While only slightly more than two-thirds of the interviewed women were directly informed about the death of their baby, just half of them were informed by a formal service provider. Meanwhile, service providers’ death notifications were usually short and did not contain explanations about events leading to the death and its causes. Simultaneously, maternal doubts and length and source of death notifications were associated with misclassification. Compared with births where the woman inquired on information on the progress of labour and the baby’s health, misclassification was less likely when the women recalled no doubts. Misclassification was more likely if death notification lasted <1 compared to 1-4 minutes. Misclassification was also more likely if a formal service provider had informed about the death compared with a family member and tended to be less likely if the death was not directly communicated to the mother.

The reported limited peripartum provider-mother interactions likely compromise maternal knowledge of the circumstances surrounding the death, possibly including the child’s time of death and vital status at birth, resulting in reduced validity of household-survey-based neonatal mortality and stillbirth estimates. While the factors we found associated with maternal misclassifications seem easily modifiable, there may be several possible explanations for their occurrence. First, time constraints of service providers may be decisive. In a qualitative study from Ethiopia, service providers explained lacking provider communication in the context of births with adverse outcomes by heavy patient loads and limited time per patient [25]. Accordingly, time constraints may explain both the limited intrapartum updates and maternal inquiries we found, as well as the shortness of service providers’ death notifications, and omissions of explanations of events leading to the death and causes of death. This may also explain why misclassification was less likely when a family member had informed about the death in comparison with a service provider as the family member may have explained the child’s passing more comprehensibly.

Socio-cultural expectations may be a further explanation. Our field observations from Guinea-Bissau suggest that conveying bad news is often regarded as a “family matter” in this setting. The circumstance that provider communications were short and incomplete may therefore reflect the socio-cultural expectation that it should not be the provider who communicates the death to the mother. Meanwhile, learning about the child death directly from a service provider may be unexpected for the woman and spark confusion, hence explaining misclassifications being associated with the service provider being the informant. Preoccupations regarding the mother’s physical and mental condition may play an additional role. In Afghanistan, Kenya, and Uganda, studies found that health workers avoided or delayed notifying the mother of an adverse birth outcome with the aim to protect her from aggravated grief and complications [22,26]. In Afghanistan, the same intentions also influenced who was notified: some service providers preferred to first disclose the child death to the woman’s companion and gave stillborn babies rather to a family member than to the mother [22]. Moreover, patient-provider relations and female empowerment may play a role. Accordingly, in Ethiopia, service providers reasoned that lacking maternal inquiries during births with adverse outcomes may be rooted in lacking maternal empowerment, education, and a “culture of not asking questions” [25]. Stigma, which has been documented to be commonly associated with adverse birth outcomes [24], may additionally compromise maternal inquiries. Similar mechanisms could be at work in the Bissau-Guinean context.

Furthermore, a low socio-cultural importance of distinguishing between adverse birth outcomes may influence the limited peripartum provider-mother interactions we found and maternal misclassifications. While in settings such as Uganda and Afghanistan, distinct rituals and burial practices depending on the child’s vital status at birth have been documented [22,33], in Guinea-Bissau, a previous qualitative study found no related distinctions [19]. Hence, knowing whether the child was stillborn or died shortly after birth may be of secondary importance to the woman, thereby entailing structurally conflicting information needs between affected women and global policymaking for newborn survival and stillbirth programmes relying on maternal information.

The wide range of potential determinants of limited peripartum provider-mother interactions highlights the importance of further contextual knowledge to enable their effective modification. Simultaneously, the dominant direction of misclassification we found underlines the general importance of improving the understanding of such mechanisms. Most discordances between HDSS and EN-INDEPTH classifications found in this study, i.e. cases, were HDSS-classified stillbirths which were classified as early neonatal deaths in the DHS-mimicking household survey (n/N = 49/63 (78%)). Hence, misclassification was not evenly bidirectionally distributed. This is a similar result to a previous study from urban Guinea-Bissau which also found an overestimation of neonatal deaths in a retrospective household survey [18]. Hence, stillbirth and early neonatal death misclassifications may not cancel each other out in household surveys, and household surveys could overestimate neonatal mortality. This poses a key challenge to global mortality tracking since the correct distinction of perinatal deaths into stillbirths or early neonatal deaths is fundamental to target and monitor preventive actions [8].

### Strengths and limitations

This study built on data collected during the retrospective EN-INDEPTH population survey mimicking DHS data collection approaches [16] and prospective mortality surveillance of BHP’s routine HDSS [31], allowing us to explore mechanisms associated with misclassification in a key source of child mortality estimates in LMICs. To do so, we treated HDSS classifications of stillbirths and early neonatal deaths as reference standard. Though we do not consider HDSS data as a perfect “gold standard” due to a residual misclassification risk, this seems warranted considering the high concordance of HDSS classifications with HNSM health facility records and this study’s case-control interview. The previous data collections enabled us to present comprehensive participant background information while keeping the present interview to a reasonable timeframe. However, our sample size was limited and prevented further investigations, e.g. regarding background factors associated with the direction of misclassification and simultaneous consideration of multiple covariates and their interactions. Further, the long recall time in the case-control interviews, and allowing others to respond on behalf of the mother in HDSS data may entail reporting inaccuracies. Moreover, due to the case-control design, no causal inference can be drawn. In addition, accidental or purposive clinical misclassification, e.g. due to poor training, high workload, lack of equipment, and avoidance of blame or extra work [9,22,25,34,35], may have modified our results. Related differences as well as differences in the socio-cultural importance assigned to the distinction between stillbirths and neonatal deaths across settings may also compromise the transferability of findings. Furthermore, as we did not include births omitted in either or both the EN-INDEPTH survey or HDSS reporting, this study cannot be directly translated into a measure of the magnitude of misclassification.

## CONCLUSIONS

While the accurate classification of perinatal deaths as stillbirths or early neonatal deaths is fundamental to target and monitor preventive action, little is known about mechanisms determining maternal misclassification in household surveys. In this study, we found limited peripartum provider-mother interactions during births with adverse outcomes in Guinea-Bissau, which likely compromise maternal knowledge of circumstances surrounding the child death and reporting validity. We also found modifiable factors associated with maternal misclassification including maternal doubts during birth and length and source of child death notifications. Moreover, our findings indicate that misclassification between stillbirths and early neonatal deaths do not cancel each other out but may rather lead to overestimation of neonatal mortality in household surveys, thereby highlighting the need for further research aiming at improving the understanding of determinants of misclassification.

## Additional material


Online Supplementary Document
