# Supplementary material for: Provider-mother interactions are associated with birth outcome misclassifications in household surveys: A case-control study in Guinea-Bissau
Source: J Glob Health. 2023 Aug 18;13:04086. doi: 10.7189/jogh.13.04086 (PMC10435094; doi:10.7189/jogh.13.04086)
Supplement: Online Supplementary Document [file jogh-13-04086-s001.pdf]

Provider-mother interactions are associated with birth outcome misclassifications in household surveys: A case-control study in Guinea-Bissau.

Supplementary material

1. Methods 2

Methods S1. Participant eligibility, record linkage, and recruitment 2

Methods S2. Consent 3

Methods S3. Source of information and classification of variables 3

Methods S4. Survey commands 10

2. Results 11

Results S1. Background characteristics of eligible and interviewed women 11

Results S2. Classification of stillbirths and early neonatal deaths across data sources 12

Results S3. Background characteristics 13

Results S4. Adjusted estimates 15

Results S5. Sensitivity analyses 27

3. Questionnaire S1 27

4. References 36

1. Methods

Methods S1. Participant eligibility, record linkage, and recruitment

Study participants were recruited among EN-INDEPTH study participants. The EN-INDEPTH study was conducted at BHP in 2017-18. It used a retrospective population survey mimicking Demographic and Health Survey (DHS) methodology to collect self-reported information on stillbirths and early neonatal deaths among women registered in the health and demographic surveillance systems (HDSS) of the Bandim Health Project (BHP) with a registered birth outcome during the past 5 years [1,2]. To identify study participants and cases and controls for the present study, we linked EN-INDEPTH records of stillbirths and early neonatal deaths with the HDSS records at the individual birth level. This involved first preparing a dataset with all births reported by women in the EN-INDEPTH interviews and all births recorded in the HDSS data. The linkage was performed for all children born to women who reported a stillbirth or early neonatal death in the EN-INDEPTH interview provided that the date of birth was within the past 5 years and during the period where the woman was under HDSS surveillance. Hence, the reported births are also likely HDSS-recorded. The linking took place in two phases. First, all EN-INDEPTH-reported births were linked to all HDSS-recorded births of the same woman to identify the HDSS-recorded birth with the date of birth closest to the EN-INDEPTH-reported birth. If there were more births matching within +/-9 months of the reported birth, the best match based on proximity of birthdate and twin number was selected (date matches). Subsequently, we identified matches based on matching siblings. Here, we allowed dates to differ, but accepted matches if an adjacent sibling was matched even if the reported and recorded year of birth differed by 1 year. This was done as birthdates of children who have died is more common to be reported incompletely [3] and with less precision [4].

378 early neonatal deaths and 278 stillbirths were reported by 611 women in the 5 years prior to the EN-INDEPTH interview. 98 of these women were not yet registered in the HDSS at the birth of their most recent early neonatal death or stillbirth and were excluded. Thus, we attempted to link 312 early neonatal deaths and 236 stillbirths among 1834 births to 513 women. A month and year of birth was reported for 459 of the perinatal deaths (84%). The remainder were classified as 15th June in the reported year in line with the HDSS coding. Using date matching (+/- 9 months), we could link 199/312 of the early neonatal deaths and 143/236 stillbirths. Further 37 early neonatal deaths and 31 stillbirths were matched based on the matching of an adjacent sibling and the reported year of birth in the survey being within +/- 1 year of the year of birth recorded in the HDSS. Thus, in total, we were able to match 236 early neonatal deaths and 174 stillbirths reported by 391 women in the EN-INDEPTH study (Figure 1).

Among the 410 matched perinatal deaths, 91 (73 early neonatal deaths and 18 stillbirths according to EN-INDEPTH data) were classified discordantly in the HDSS data. Two women had two discordantly classified birth outcomes. Thus, 89 cases and 302 controls were eligible for the case-control study. These records were linked to the current HDSS data to extract a list of the women's last known address. To recruit participants, BHP interviewers visited each eligible woman.

Methods S2. Consent

Provided that the HDSS data collector known by the mothers had obtained consent to a visit, a specially trained fieldworker of the case-control study team visited the household. The team member provided information on the objective of the study and requested the mother to participate in an interview lasting up to 1 hour. The team member emphasized that the participation was voluntary, that there was no direct benefit of participating and that information would be considered confidential. Furthermore, information was provided that the woman could stop the interview at any time and that she did not have to respond to all questions. Provided the woman accepted to participate, she would be asked to sign a consent form. For women who could not read and/or write, she was asked to summon a bystander at her choice who acted as an independent witness and testified the consent process while the woman's consent was documented by fingerprint. If the interviewer was unable to locate a woman at her address at first attempt in the rural area, one re-attempt was made. In the urban area, a minimum of two re-attempts were made.

Methods S3. Source of information and classification of variables

Table S1: Classification of vital status at birth across data sources.

Data source	Early neonatal death	Stillbirth
HDSS	Registration of a pregnancy outcome (birth questionnaire): Was the child born dead or alive? Probing question: Did the child cry?	
EN-INDEPTH: FBH+	Reported birth in the woman's birth history with reported death within the first week of life.	Probe on stillbirths after recording the woman's birth history: Have you ever had a pregnancy that miscarried, was aborted, or ended in a stillbirth?
EN-INDEPTH: FPH	Reported pregnancy in the woman's pregnancy history with reported death within the first week of life and affirmation of the probe on a reported pregnancy outcome: Did this baby cry, move, or breathe when it was born?	Reported pregnancy in the woman's pregnancy history with reported death and no affirmation of the listed probe on the pregnancy outcome.
HNSM	Classification as "liveborn" in the birth record.	Classification as "stillborn" in the birth record.
Case-control interview	Affirmation of any sign of life based on the following questions:<br>1. Immediately after you gave birth, did you personally see, hear, or feel your baby showing any of the following signs of life?<br>a. Baby was crying<br>b. Baby was moving<br>c. Baby was breathing<br>d. Baby had heartbeat<br>2. Were you told whether or not the baby was crying? [If yes] Did your baby cry?<br>3. Were you told whether or not the baby was moving? [If yes] Did your baby move?<br>4. Were you told whether or not the baby was breathing? [If yes] Did your baby breath?<br>5. Were you told whether or not the baby had a heartbeat? [If yes] Did your baby have a heartbeat?	No sign of life reported based on the listed questions.

EN-INDEPTH: EN-INDEPTH study conducted in 2017-18 [2]; FBH+: Full birth history survey with probes on pregnancy loss; FPH: Full pregnancy history survey; HDSS: Health and Demographic Surveillance System; HNSM: Records from the National Hospital Simão Mendes.

Table S2: Source of information and classification of background variables

	Variable	Definition	Source	Scale
1	**Residency[1]**	Place of residence of the woman	HDSS	Categorical: urban HDSS area/rural HDSS area
2	**Maternal age**	Maternal age at the date of birth of the child we interviewed about	Calculated based on EN-INDEPTH data (maternal age at EN-INDEPTH interview and date of birth)	Categorical:<20, 20-29, 30-39, and 40+
3	**Maternal schooling[2]**	Reported level of schooling attained by the woman at the time of the EN-INDEPTH interview	EN-INDEPTH	Categorical: no schooling, primary, secondary, higher
4	**Parity[3]**	Previous pregnancies prior to the pregnancy we interviewed about, regardless of pregnancy outcome	Case-control interview	Categorical: primigravida or multipara (1+ prior births), don't know
5	**Prior adverse birth outcomes[3]**	Experience of a stillbirth or early neonatal death prior to the birth we interviewed about	Case-control interview	Categorical: yes, no, don't know
6	**Wealth quintile**	Socioeconomic classification of the woman based on recorded household assets	EN-INDEPTH interview	Categorical: poorest, 2, 3, 4, richest
7	**Ethnicity**	Woman's ethnicity	EN-INDEPTH interview	Categorical: Balante (incl. Balante Mane), Fula/Mandinga, Mancanha/Manjaco, Pepel, other/mixed (incl. Beafada, Bijago, Felupe, Geba, Saracole)
8	**Survey module**	Survey module used in EN-INDEPTH interview	EN-INDEPTH interview	Categorical: FBH+, FPH
9	**Recall HDSS[1]**	Recall length in the HDSS interview	Calculated based on date of first HDSS-interview after the pregnancy and HDSS-recorded date of birth	Categorical: ≤ median, > median
10	**Recall EN-INDEPTH[1]**	Recall length in the EN-INDEPTH interview	Calculated based on date of EN-INDEPTH interview and EN-INDEPTH-recorded date of birth)	Categorical: ≤ median, > median
11	**Recall case-control interview**	Recall length in the case-control interview	Calculated based on date of case-control interview and EN-INDEPTH-recorded date of birth)	Categorical: ≤ median, > median
12	**Proxy Reporting[1]**	Provider of HDSS responses	HDSS	Categorical: mother responded, somebody else responded

13	**Number of ANCs obtained**	Number of ANC consultations obtained	Case-control interview	Categorical: none, 1-3, 4-7, ≥8, don't know
14	**ANC diagnostics provided**	Diagnostics obtained during ANC – 3 diagnostics inquired: (i) blood pressure measured, (ii) urine sample taken, (iii) blood sample taken	Case-control interview	Categorical: none (none or don't know), some (1-2 diagnostics obtained), comprehensive (all 3 diagnostics obtained)
15	**ANC counselling provided**	Counselling obtained during ANC – counselling on 3 topics inquired: (i) advice on nutrition, (ii) advice on danger signs, (iii) action to be taken on danger signs (return to health facility)	Case-control interview	Categorical: none (none or don't know), some (counselling on 1-2 topics obtained), comprehensive (counselling on all 3 topics obtained)
16	**Maternal risk factors during pregnancy**	Maternal risk factors experienced during pregnancy, labour, or delivery – 14 risk factors inquired: (i) convulsions, (ii) high blood pressure, (iii) some vaginal bleeding, (iv) significant vaginal bleedings, (v) severe lack of blood or pallor and shortness of breath, (vi) diabetes, (vii) severe headache, (viii) blurred vision, (ix) significant weakness, (x) significant abdominal pain aside from labour, (xi) rapid/difficulties breathing, (xii) puffy face, (xiii) fever, (xiv) smelly vaginal discharge	Case-control interview	Categorical: none (none or don't know), 1 reported, 2-5 reported, >5 reported
17	**Health professional informed that pregnancy was high-risk**	Mother had been informed about possible dangers of the pregnancy related to the baby's health prior to the birth	Case-control interview	Categorical: yes, mentioned that the baby may have minor health problems, yes, mentioned the baby may have serious health problems (including it may be stillborn), no, don't know
18	**Facility birth[1]**	Place of birth	Case-control interview	Categorical: yes, no, don't know
19	**Birth attendant**	Qualification of the person who assisted the birth	Case-control interview	Categorical: skilled birth attendant (medical doctor, midwife, nurse or unspecified provider at health facility; including 'don't know' if the place of birth was a health facility), traditional birth attendant, other (a family member or another person not considered a birth attendant assisted or the mother gave birth alone), don't know
20	**Referred to HF**	Women had been referred to the place of birth	Case-control interview	Categorical: yes, no, don't know
21	**Type of birth[4]**	Type of delivery	Case-control interview	Categorical: vaginally, instrumental vaginally, C-Section, don't know
22	**Duration of labour and birth**	Time of labour and delivery	Case-control interview	Categorical: less than 6 hours, 6-11 hours, 12-18 hours, more than 18 hours, don't know

23	**Time of birth**	Time of day when the baby was born	Case-control interview	Categorical: daytime (8am-4pm), evening (4pm-12am), late night (12am-8am), don't know
24	**Multiple gestation**	How many babies were delivered	Case-control interview	Categorical: one baby, more than one baby, don't know
25	**Intrapartum complications**[5]	Intrapartum complications experienced: breech or transverse position, umbilical prolapse, cord around neck, excessive bleeding, others	Case-control interview	Categorical: none (none or don't know), 1 reported, >1 reported
26	**Baby moved before birth**	Was the baby moving the last few days before birth	Case-control interview	Categorical: yes, no, don't know
27	**Mother noticed birth injury**	Were there any bruises or signs of injury on the baby's body at birth	Case-control interview	Categorical: yes, no, don't know
28	**Place of baby's death**[6]	Where did the baby die	Case-control interview	Categorical: hospital of delivery, other health facility, home, way to a health facility/hospital, other, don't know
29	**Time of baby's death**	When did the baby pass	Case-control interview	Categorical: before birth, immediately after birth, within one week after birth, more than one week after birth, don't know

Case-control interview: Interview of the present case-control study; EN-INDEPTH: EN-INDEPTH study conducted in 2017-18 [2]; HDSS: Health and Demographic Surveillance System.

[1] Included in adjusted analyses.

[2] Included in adjusted analyses; categories (reduced): no schooling, primary, secondary/higher.

[3] Included in adjusted analyses; categories (combined): primigravida, multipara: yes (prior adverse outcome), multipara: no (prior adverse outcome).

[4] Included in adjusted analyses; categories (reduced): C-Section, no C-Section.

[5] Included in adjusted analyses; categories (reduced): none, >=1.

[6] Included in adjusted analyses; categories (reduced): hospital of delivery, other health facility, home, way to a health facility/hospital, other.

Table S3: Source of information and classification of independent variables

	Variable	Definition	Source	Scale
1	**Updates obtained on baby's/women's health and progress of labour**	Updates obtained during delivery – updated on 5 topics inquired: (i) whether the baby was alive or deceased, (ii) whether the baby will survive, (iii) progress of labour, (iv) complications affecting maternal health during labour, (v) complications affecting baby's health during labour	Case-control interview	Categorical: none (none or don't know), any (1 reported), more comprehensive (2-5 reported)
2	**Mother was worried about baby's health**	Was the mother worried about the health of the baby when going into delivery	Case-control interview	Categorical: no, yes (little/average), yes (very/extreme), don't know
3	**Mother inquired progress of labour**	Did the mother ask the birth attendant any questions about the progress of labour during delivery	Case-control interview	Categorical: yes (asked questions), no (was unable), no (no doubts recalled), don't know
4	**Mother inquired baby's health during labour**	Did the mother ask the birth attendant any questions about how the baby was doing during delivery	Case-control interview	Categorical: yes (asked questions), no (was unable), no (no doubts recalled), don't know
5	**Mother saw baby postpartum**	After the delivery, was the baby close enough to the mother to see, hear, or feel the baby	Case-control interview	Categorical: yes, no, don't know
6	**Death was directly communicated to the mother**	Was the death directly communicated to the mother	Case-control interview	Categorical: yes, no, don't know
7	**First person who communicated the death to the mother**	Who was the first person who told the mother that the baby had died (only if the women recalled that the death was directly communicated)	Case-control interview	Categorical: formal service provider, informal service provider, family member/partner, other
8	**Mother's familiarity with the provider**	How many times had the mother previously visited the person (skilled or traditional birth attendant) who communicated the death to the mother (only asked if the mother was directly informed by a skilled or traditional birth attendant)	Case-control interview	Categorical: none, 1 previous encounter, ≥2 previous encounters, don't know
9	**Mother's location when learning about death**	Where was the mother when she was told that the baby had passed	Case-control interview	Categorical: delivery room, maternity ward, at home, another place in the health facility, another place outside the health facility, don't know
10	**Presence of other people**	Who else was in the room when the mother was told the baby had passed	Case-control interview	Categorical: the woman was alone, only other unknown people, family only, family and unknown people, don't know

**11**	**Language spoken**	What language did the person (skilled or traditional birth attendant) who communicated the death to the mother speak (only asked if the mother was directly informed by a skilled or traditional birth attendant)	Case-control interview	Categorical: Guinea-Bissau Creole, Portuguese, other, don't know
**12**	**Comprehensibility of information provided**	Did the person talk about the death of the baby in a way that the mother could understand (only asked if the mother was directly informed by a skilled or traditional birth attendant)	Case-control interview	Categorical: no (they said a vague statement that might have suggested the baby died), no (they used medical words the mother did not understand), yes (but the mother had to ask for more information), yes (the mother understood them immediately), don't know
**13**	**Comprehensibility of events leading to death**	Did the person communicate the events that led to the death in a way the mother could understand (only asked if the mother was directly informed by a skilled or traditional birth attendant)	Case-control interview	Categorical: no (they did not mention the events leading up to death), no (they were uncertain of the events leading up to death), no (they used medical words the mother did not understand), yes (but the mother had to ask for more information), yes (the mother understood them immediately), don't know
**14**	**Comprehensibility of causes of death**	Did the person communicate the cause of death in a way the mother could understand (only asked if the mother was directly informed by a skilled or traditional birth attendant)	Case-control interview	Categorical: no (they did not mention the cause of death), no (they were uncertain of the cause of death), no (they used medical words the mother did not understand), yes (but the mother had to ask for more information), yes (the mother understood them immediately), don't know
**15**	**Completeness of information**	Did this person tell all the information the mother wanted to know (only asked if the mother was directly informed by a skilled or traditional birth attendant)	Case-control interview	Categorical: yes (they told the mother what she needed to know), no (they told the mother too much information, no (they did not tell the mother enough information), don't know
**16**	**Timing of information provision**	How soon after delivery did this conversation about the baby's death occur (only asked if the mother was directly informed by a skilled or traditional birth attendant)	Case-control interview	Categorical: immediately postpartum, 1-2hrs postpartum, 3-24hrs postpartum, >24hrs postpartum, don't know
**17**	**Duration of information provision**	How long did this person spend talking to the mother about the baby's passing (only asked if the mother was directly informed by a skilled or traditional birth attendant)	Case-control interview	Categorical: <1min, 1-4min, >5min, don't know
**18**	**Mother recalls being comforted by the provider**	Does the mother recall that the person tried to comfort her and/or acknowledged her suffering (only asked if the mother was directly informed by a skilled or traditional birth attendant)	Case-control interview	Categorical: yes, no (no or don't know)
**19**	**Mother recalls that the provider was dismissive**	Does the mother recall that the person said hurtful comments and/or blamed her for the death and/or tried to hide or suppress	Case-control interview	Categorical: yes, no (no or don't know)

		the mother's grief (only asked if the mother was directly informed by a skilled or traditional birth attendant)		
**20**	**Mother recalls that the provider did not provide any counselling**	Does the mother recall that the provider stayed or left without counselling or saying or doing more (only asked if the mother was directly informed by a skilled or traditional birth attendant)	Case-control interview	Categorical: yes, no (no or don't know)
**21**	**Informant paid full attention to mother while informing about baby's death**	Besides concentrating on the mother, was this person doing other things while telling the mother about the death of the baby	Case-control interview	Categorical: they paid full attention to the mother, they were distracted, don't know

Case-control interview: Interview of the present case-control study.

Methods S4. Survey commands

In BHP's HDSS data collection, participants are sampled based on their residency in a defined geographical area (rural HDSS: selected village cluster across all rural regions of Guinea-Bissau [5]; urban HDSS: a defined part of the capital city Bissau [6]). To account for possible cluster effects based on residency, we used Stata survey commands (svy). In the main analyses, we used the default method for the estimation of standard errors, Taylor linearization, and the default reporting of missing values for the standard errors when a stratum with one sampling unit was encountered. Since the majority of study participants reside in one single sampling unit (urban HDSS), in sensitivity analyses, we altered the single sampling unit criterium to certainty, scaled, and centred estimation, respectively.

2. Results

Results S1. Background characteristics of eligible and interviewed women

Table S4: Comparison of background characteristics between interviewed and not interviewed eligible women.

	CC Participants[1]	EN-INDEPTH only[2]	Total	P-value[3]
**N (%)**	278 (54)	235 (46)	513 (100)	
**Residency**				0.921
rural, n (%)	85 (31)	71 (30)	156 (30)	
urban, n (%)	193 (69)	164 (70)	357 (70)	
**Maternal age**				<0.001
<20 years, n (%)	39 (14)	60 (26)	99 (19)	
20-29 years, n (%)	131 (47)	136 (58)	267 (52)	
30-39 years, n (%)	97 (35)	37 (16)	134 (26)	
40+ years, n (%)	11 (4)	2 (1)	13 (3)	
**Highest level of school attained**				0.177
No schooling, n (%)	96 (35)	71 (30)	167 (33)	
Primary, n (%)	79 (28)	78 (33)	157 (31)	
Secondary, n (%)	103 (37)	86 (37)	189 (37)	
**Wealth quintile urban[4]**				0.291[5]
poorest, n (%)	44 (23)	49 (30)	93 (26)	
2, n (%)	64 (33)	41 (25)	105 (29)	
3, n (%)	53 (27)	46 (28)	99 (28)	
4, n (%)	32 (17)	28 (17)	60 (17)	
**Wealth quintile rural[6]**				0.391
4, n (%)	23 (27)	15 (21)	38 (24)	
richest, n (%)	62 (73)	56 (79)	118 (76)	
**Ethnicity**				0.297
Balante, n (%)	31 (11)	34 (14)	65 (13)	
Fula/Mandinga, n (%)	82 (29)	78 (33)	160 (31)	
Mancanha/Manjaco, n (%)	45 (16)	33 (14)	78 (15)	
Pepel, n (%)	66 (24)	51 (22)	117 (23)	
Other/Mixed, n (%)	54 (19)	39 (17)	93 (18)	

CC: Case-control interview; EN-INDEPTH: EN-INDEPTH study conducted in 2017-18 [2].
[1] Women who were interviewed in the present case-control study.
[2] Women who were potentially eligible for this study as they participated in the EN-INDEPTH study and were members of the Bandim Health Project's health and demographic surveillance systems (HDSS) at the time of the reported birth outcome but were not interviewed in the present case-control study. This was the case when the birth outcome could not be matched with an HDSS birth record, the women migrated out of the HDSS area, died before the interview or was temporarily absent at the dates when interviews were attempted (cf. Figure 1).
[3] Chi-Squares adjusted for possible cluster effects associated with respondents' residency.
[4] Among all women with urban residence.
[5] Not adjusted for residency since all urban residents belong to one single stratum.
[6] Among all women with rural residence.

Results S2. Classification of stillbirths and early neonatal deaths across data sources

Table S5: Classification of stillbirths vs. early neonatal deaths across data sources.

Data Source	Reported Outcome	N	Matching classification									
			HDSS		EN-INDEPTH: FBH+		EN-INDEPTH: FPH		HNSM[1]		CC	
			n	%	n/N	%	n/N	%	n/N	%	n	%
HDSS	ENND	130			52/58	90%	64/72	89%	31/36	86%	105	81%
	SB	148			54/79	68%	45/69	65%	54/59	92%	123	83%
EN-INDEPTH: FBH+	ENND	77	52	68%					17/26	65%	57	74%
	SB	60	54	90%					19/20	95%	54	90%
EN-INDEPTH: FPH	ENND	88	64	73%					17/29	59%	64	73%
	SB	53	45	85%					19/20	95%	50	94%

CC: Case-control interview; EN-INDEPTH: EN-INDEPTH study conducted in 2017-18 [2]; ENND: early neonatal death; FBH+: Full birth history survey with probes on pregnancy loss; FPH: Full pregnancy history survey; HDSS: Health and Demographic Surveillance System; HNSM: Records from the National Hospital Simão Mendes. SB: Stillbirth.
Heated shading indicates the extent of concordance between two data sources. The greener, the more concordance; the redder, the less concordance.
[1] A total of 104/278 women reported to have given birth at HNSM - matching records were identified for 95/104.

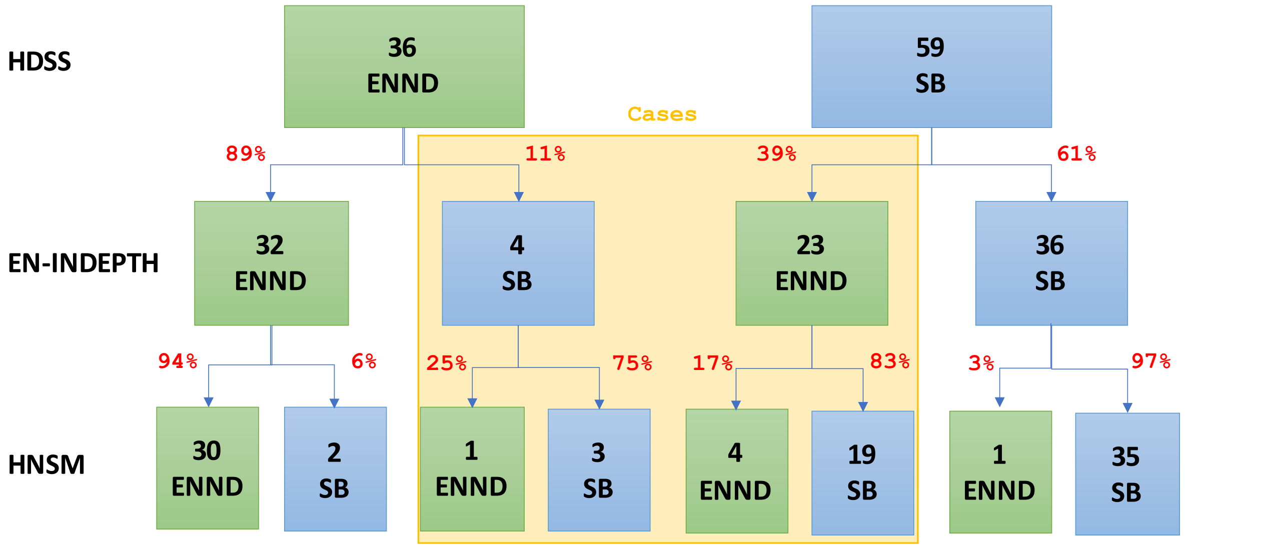

EN-INDEPTH: EN-INDEPTH study conducted in 2017-18 [2]; ENND: early neonatal death; HDSS: Health and Demographic Surveillance System; HNSM: Records from the National Hospital Simão Mendes; SB: Stillbirth.

Figure S1: Classification of stillbirths and early neonatal deaths across data sources, restricted to the subsample of women who gave birth at the National Hospital Simão Mendes.

Results S3. Background characteristics

Table S6: Antenatal care characteristics.

	Cases	Controls	Total	P-value[1]
**N (%)**	63 (23)	215 (77)	278 (100)	
**Number of ANCs obtained**				0.790
None, n (%)	0 (0)	2 (1)	2 (1)	
1-3, n (%)	6 (10)	25 (12)	31 (11)	
4-7, n (%)	38 (60)	129 (60)	167 (60)	
>=8, n (%)	8 (13)	30 (14)	38 (14)	
Unknown, n (%)[2]	11 (17)[2]	29 (13)[2]	40 (14)[2]	
**ANC diagnostics provided**				0.756
None, n (%)	2 (3)	5 (2)	7 (3)	
Some, n (%)	7 (11)	30 (14)	37 (13)	
Comprehensive, n (%)	54 (86)	180 (84)	234 (84)	
**ANC counselling provided**				0.670
None, n (%)	11 (17)	37 (17)	48 (17)	
Some, n (%)	23 (37)	88 (41)	111 (40)	
Comprehensive, n (%)	29 (46)	90 (42)	119 (43)	
**Maternal risk factors during pregnancy**				0.706
None, n (%)	11 (17)	30 (14)	41 (15)	
1, n (%)	10 (16)	43 (20)	53 (19)	
2-5, n (%)	32 (51)	110 (51)	142 (51)	
>=6, n (%)	10 (16)	32 (15)	42 (15)	
**Health professional informed that pregnancy was a risk[3]**				0.015
No, n (%)	46 (82)	147 (84)	193 (83)	
Yes, minor problems mentioned, n (%)	5 (9)	5 (3)	10 (4)	
Yes, serious problems mentioned, n (%)	3 (5)	21 (12)	24 (10)	
Unknown, n (%)[2]	2 (4)[2]	3 (2)[2]	5 (2)[2]	

ANC: Antenatal care consultations.

[1] Chi-Squares adjusted for possible cluster effects associated with respondents' residency.

[2] Shown for completeness but excluded from statistical comparison.

[3] Among all women who gave birth at a health facility (n=232).

Table S7: Survey characteristics.

	Cases	Controls	Total	P-value[1]
**N (%)**	63 (23)	215 (77)	278 (100)	
**Survey module**				0.988
FBH+, n (%)	31 (49)	106 (49)	137 (49)	
FPH, n (%)	32 (51)	109 (51)	141 (51)	
**Recall length HDSS[2]: median 0.6 months (IQR 0.1-1.2)**				0.053
<= median, n (%)	29 (46)	116 (54)	145 (52)	
> median, n (%)	34 (54)	98 (46)	132 (48)	
**Recall length EN-INDEPTH: median 26 months (IQR 9-43)**				0.748
<= median, n (%)	33 (52)	107 (50)	140 (50)	
> median, n (%)	30 (48)	108 (50)	138 (50)	
**Recall length CC: median 74 months (IQR 59-92)**				0.427
<= median, n (%)	35 (56)	104 (48)	139 (50)	
> median, n (%)	28 (44)	111 (52)	139 (50)	
**Proxy reporting[3]**				0.044
Mother reported, n (%)	44 (79)	175 (86)	219 (85)	
Somebody else reported, n (%)	12 (21)	28 (14)	40 (15)	

CC: Case-control interview; EN-INDEPTH: EN-INDEPTH study using household survey mimicking Demographic and Health Survey methodology [2]; FBH+: Full birth history survey; FPH: Full pregnancy history survey; HDSS: Health and demographic surveillance system of the Bandim Health Project.

[1] Chi-Squares adjusted for possible cluster effects associated with respondents' residency.

[2] 1 missing value (original HDSS record could not be found).

[3] 19 missing values (information has not been recorded).

Results S4. Adjusted estimates

Table S8: Background factors investigated as potential confounders.

	Cases	Controls	OR (95% CI)[1]
**N (%)**	63 (23)	215 (77)	
**Residency**			
Rural, n (%)	22 (35)	63 (29)	ref.
Urban, n (%)	41 (65)	152 (71)	0.77 (0.46-1.30)
**Highest level of school attained**			
No schooling, n (%)	24 (38)	72 (33)	ref.
Primary, n (%)	19 (30)	60 (28)	0.95 (0.40-2.23)
Secondary or higher, n (%)	20 (32)	83 (39)	0.72 (0.45-1.17)
**Prior adverse birth outcome[2]**			
Primigravida, n (%)	19 (30)	52 (24)	1.54 (1.09-2.18)
Yes, n (%)	16 (25)	44 (21)	1.53 (0.96-2.44)
No, n (%)	28 (44)	118 (55)	ref.
**Recall length HDSS[3]**			
<= median, n (%)	29 (46)	116 (54)	ref.
> median, n (%)	34 (54)	98 (46)	1.39 (0.99-1.94)
**Recall length EN-INDEPTH**			
<= median, n (%)	33 (52)	107 (50)	ref.
> median, n (%)	30 (48)	108 (50)	0.90 (0.47-1.72)
**Facility birth**			
Yes, n (%)	56 (89)	176 (82)	ref.
No, n (%)	7 (11)	39 (18)	0.56 (0.26-1.22)
**Mother recalls delivery by C-section**			
Yes, n (%)	10 (16)	33 (15)	ref.
No, n (%)	53 (84)	182 (85)	0.96 (0.61-1.51)
**Intrapartum complications**			
None reported, n (%)	29 (46)	104 (48)	ref.
1 reported, n (%)	34 (54)	111 (52)	1.10 (0.76-1.59)
**Place of death[4]**			
HF of delivery, n (%)	49 (79)	135 (63)	ref.
Other HF, n (%)	1 (2)	24 (11)	0.11 (0.01-1.56)
Home, n (%)	11 (18)	46 (21)	0.66 (0.25-1.72)
Other, n (%)	1 (2)	9 (4)	0.31 (0.07-1.30)
**Proxy Reporting[5]**			
Mother reported, n (%)	44 (79)	175 (86)	ref.
Somebody else reported, n (%)	12 (21)	28 (14)	1.70 (1.01-2.88)

EN-INDEPTH: EN-INDEPTH study using household survey mimicking Demographic and Health Survey methodology [2]; HDSS: Health and demographic surveillance system of the Bandim Health Project.

[1] Adjusted for possible cluster effects associated with respondents' residency.

[2] "Don't know" has been dropped from this analysis, therefore 1 missing value (cf. Table 1).

[3] 1 missing value (original HDSS record could not be found).

[4] "Don't know" has been dropped from this analysis, therefore 2 missing values (cf. Table 2).

[5] 19 missing values (information has not been recorded).

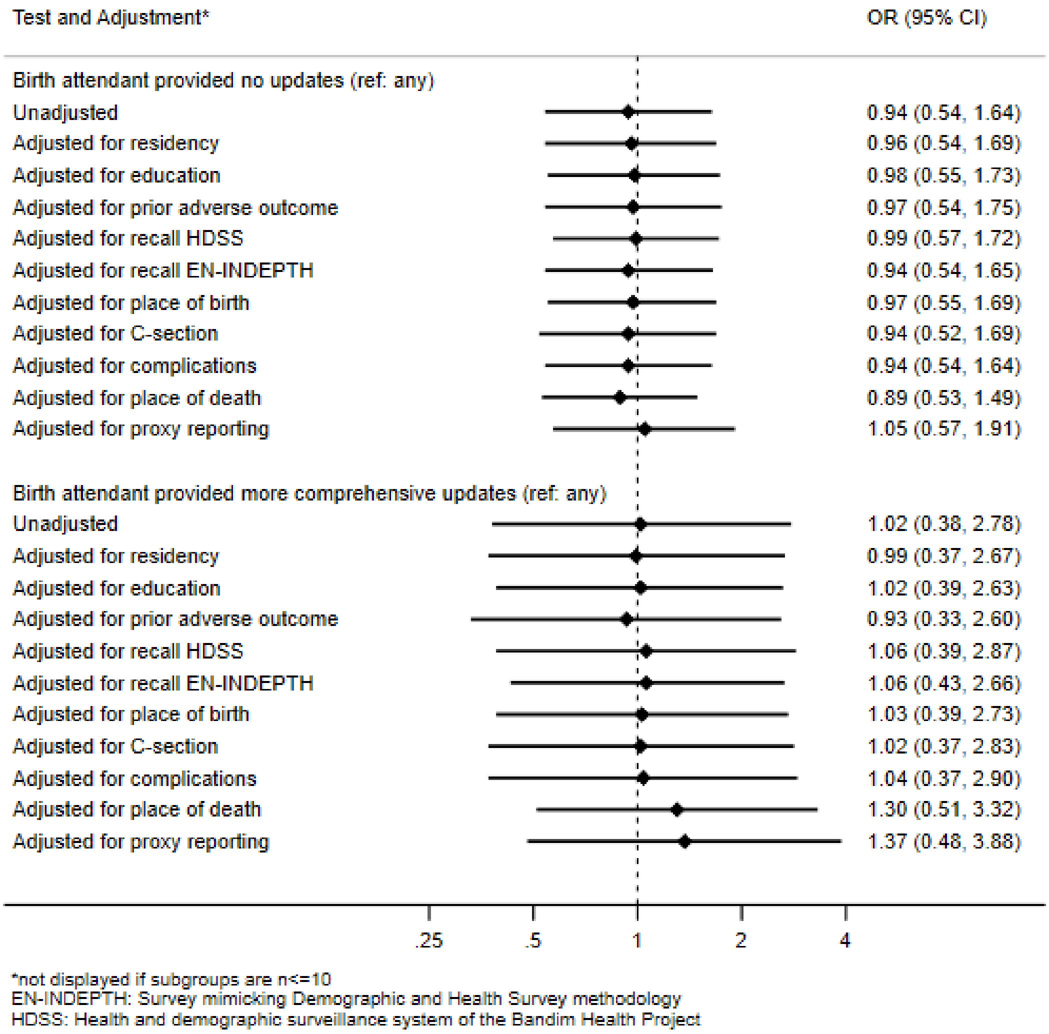

Figure S2: Multivariate regressions: Birth attendant provided updates on the progress of labour and the health of the baby and the mother during delivery.

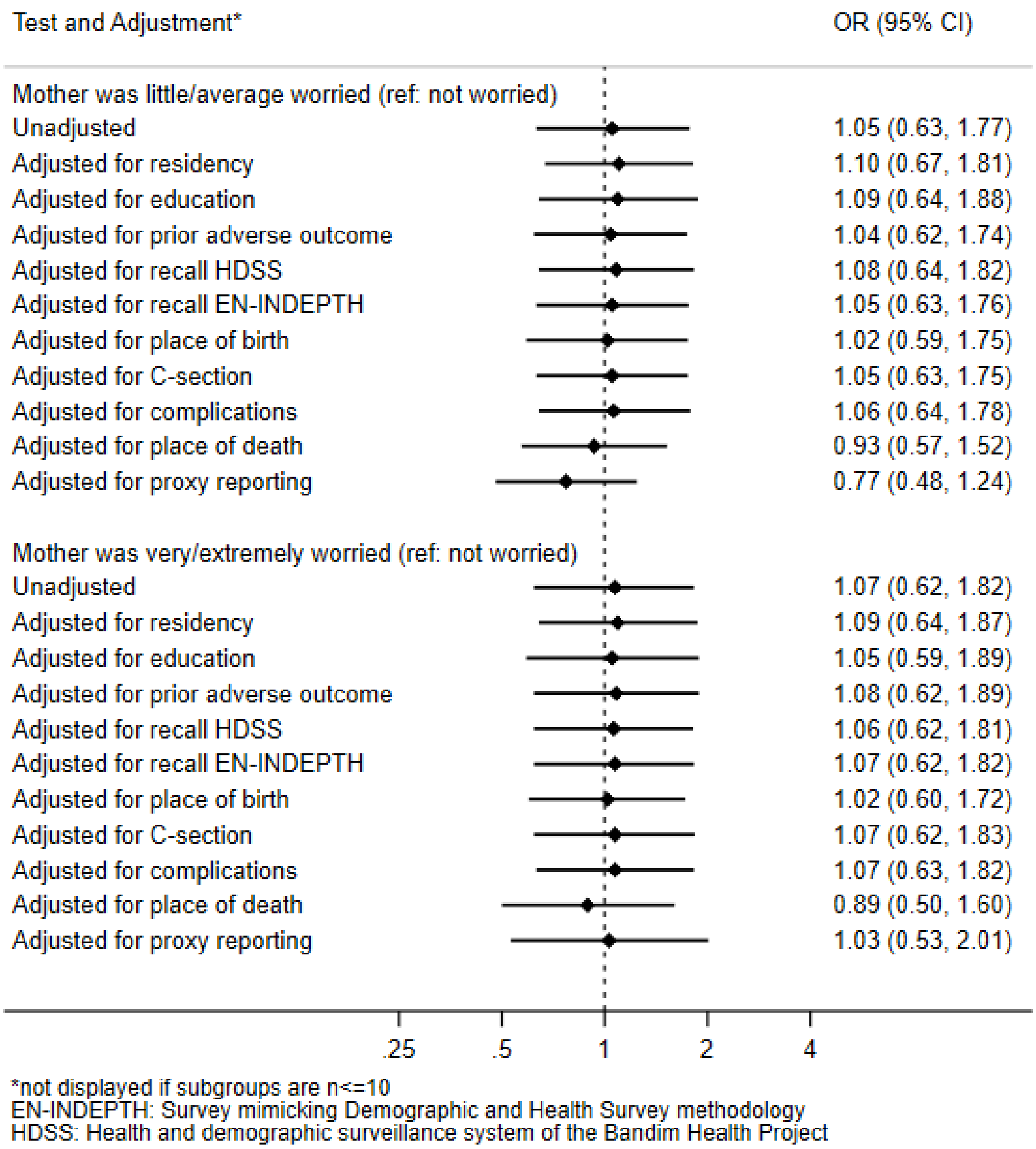

Figure S3: Multivariate regressions: Maternal worries about baby's health.

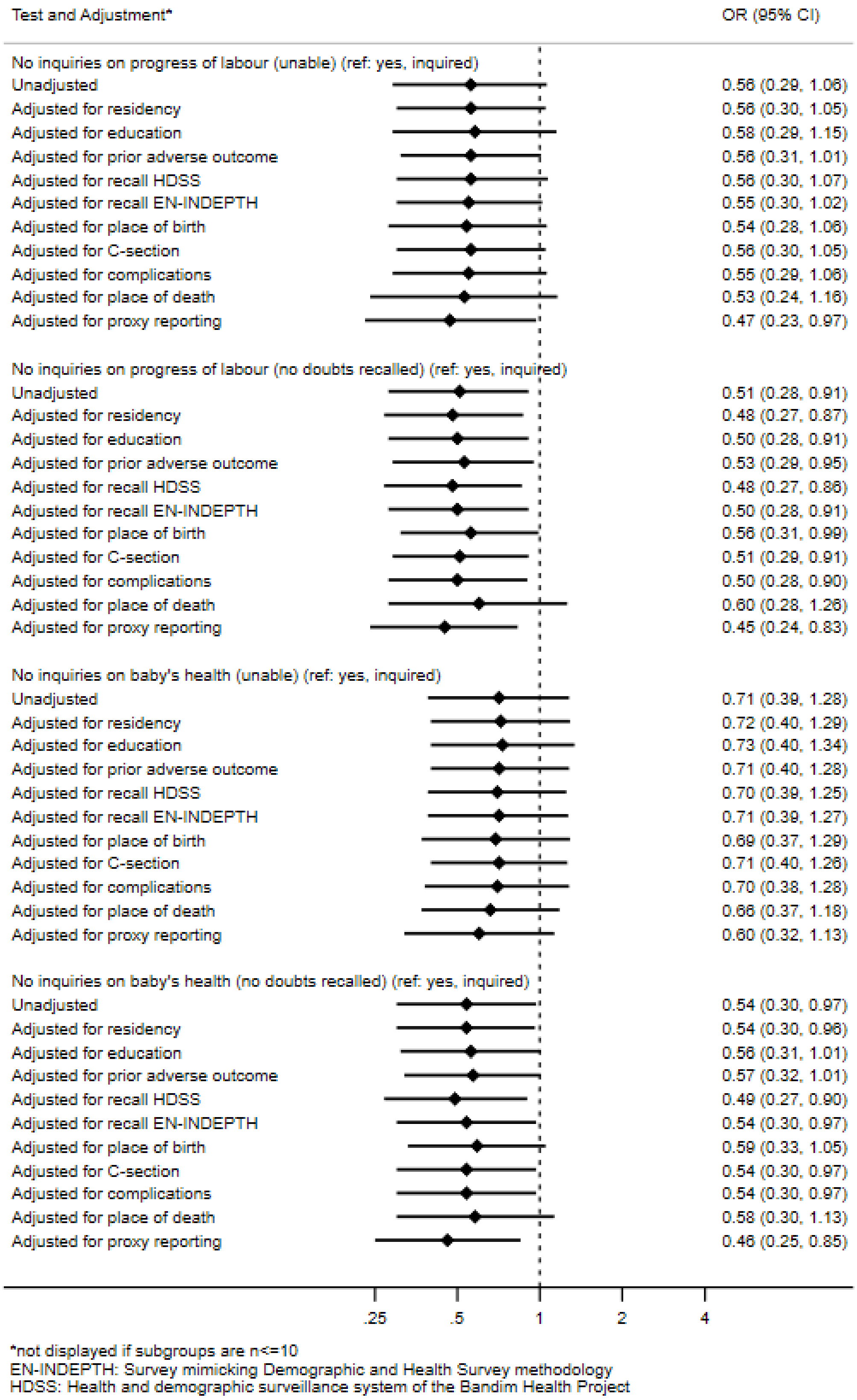

Figure S4: Multivariate regressions: Maternal inquiries on progress of labour/baby's health during labour.

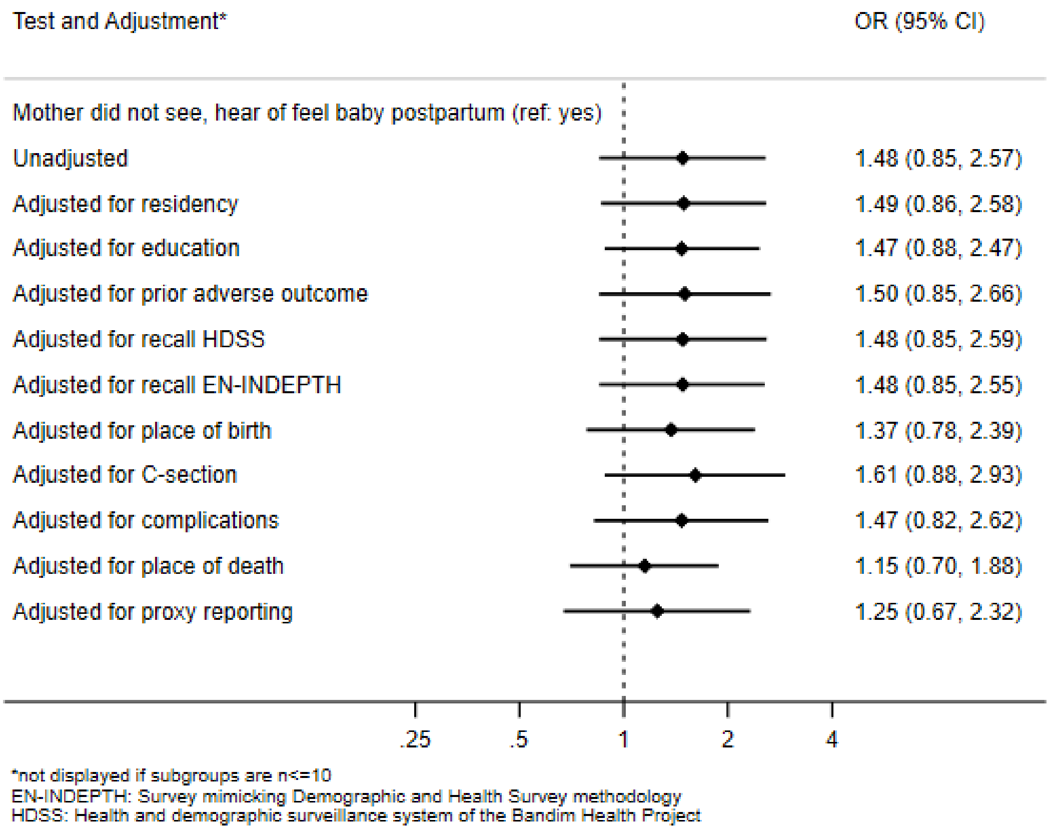

Figure S5: Multivariate regressions: Mother saw, heard of felt baby postpartum.

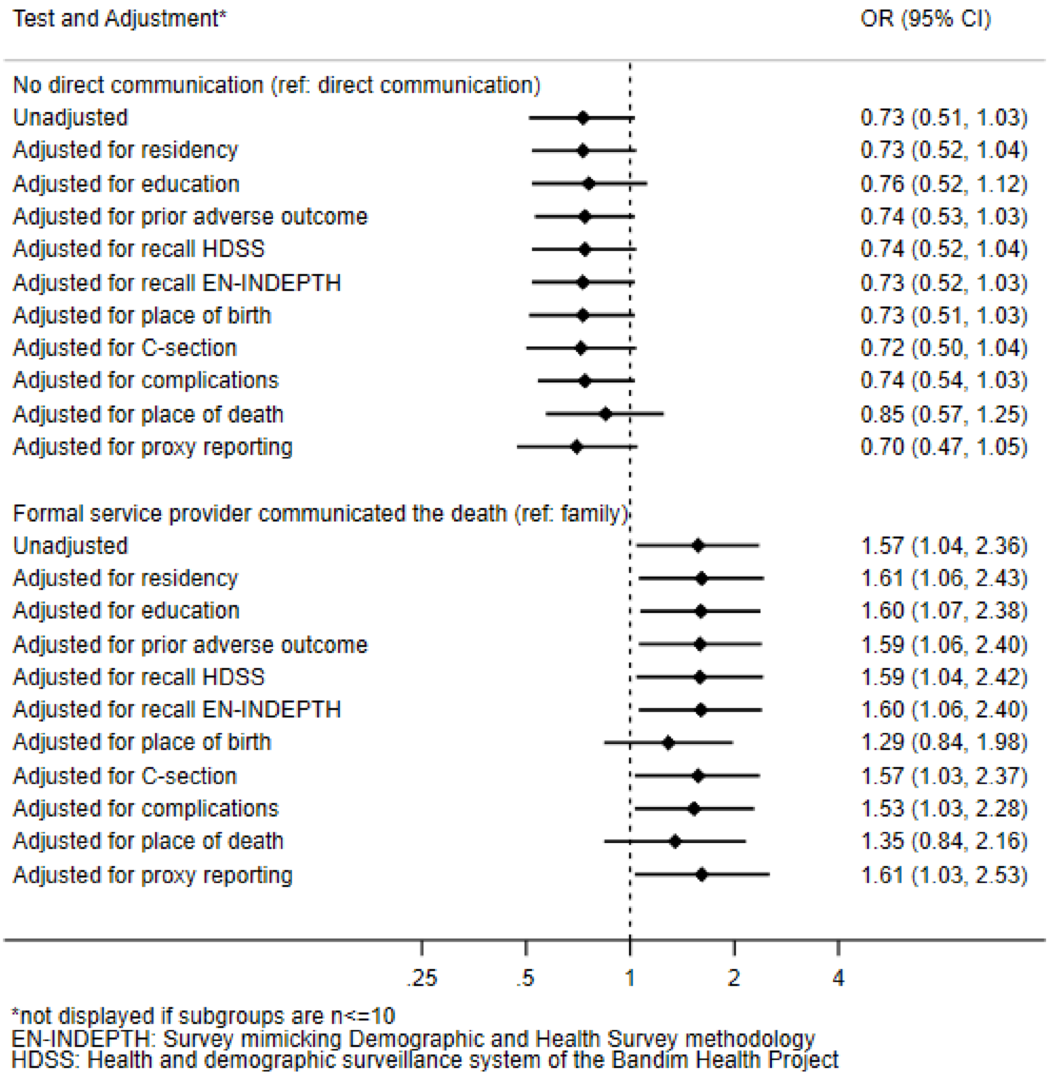

Figure S6: Multivariate regressions: Person communicating the death to the mother.

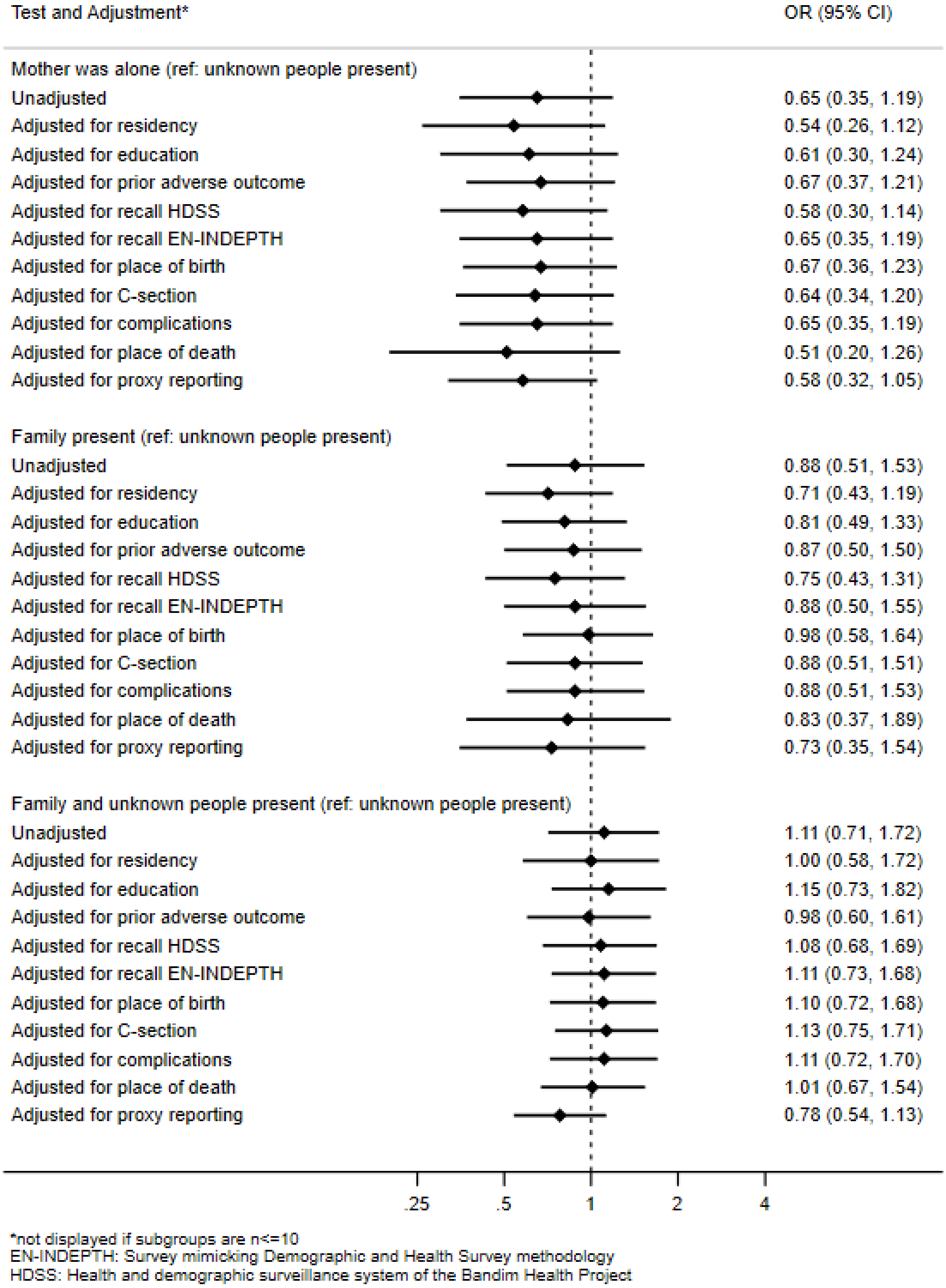

Figure S7: Multivariate regressions: Presence of other people when learning about the death.

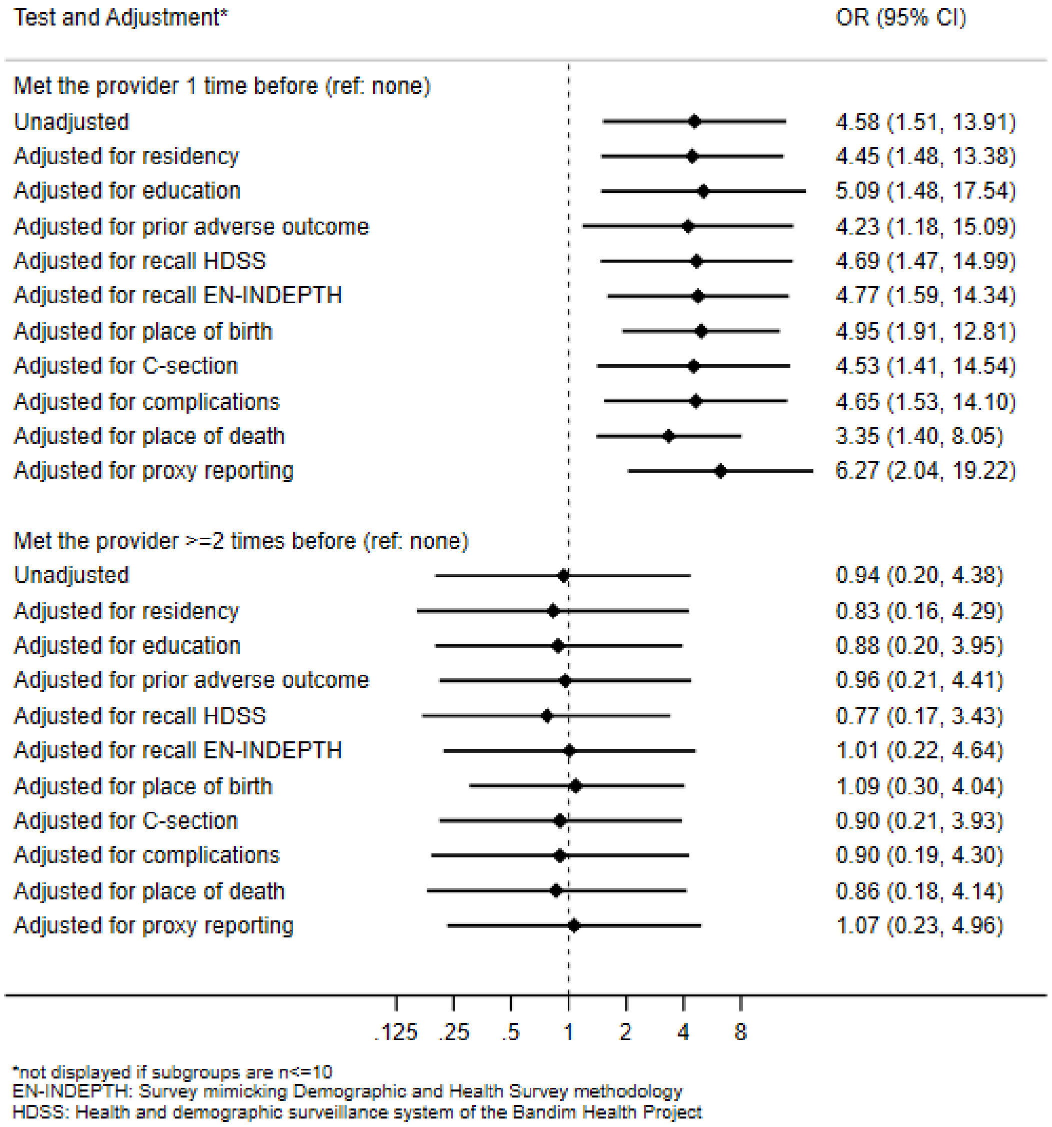

Figure S8: Multivariate regressions: Familiarity with the service provider.

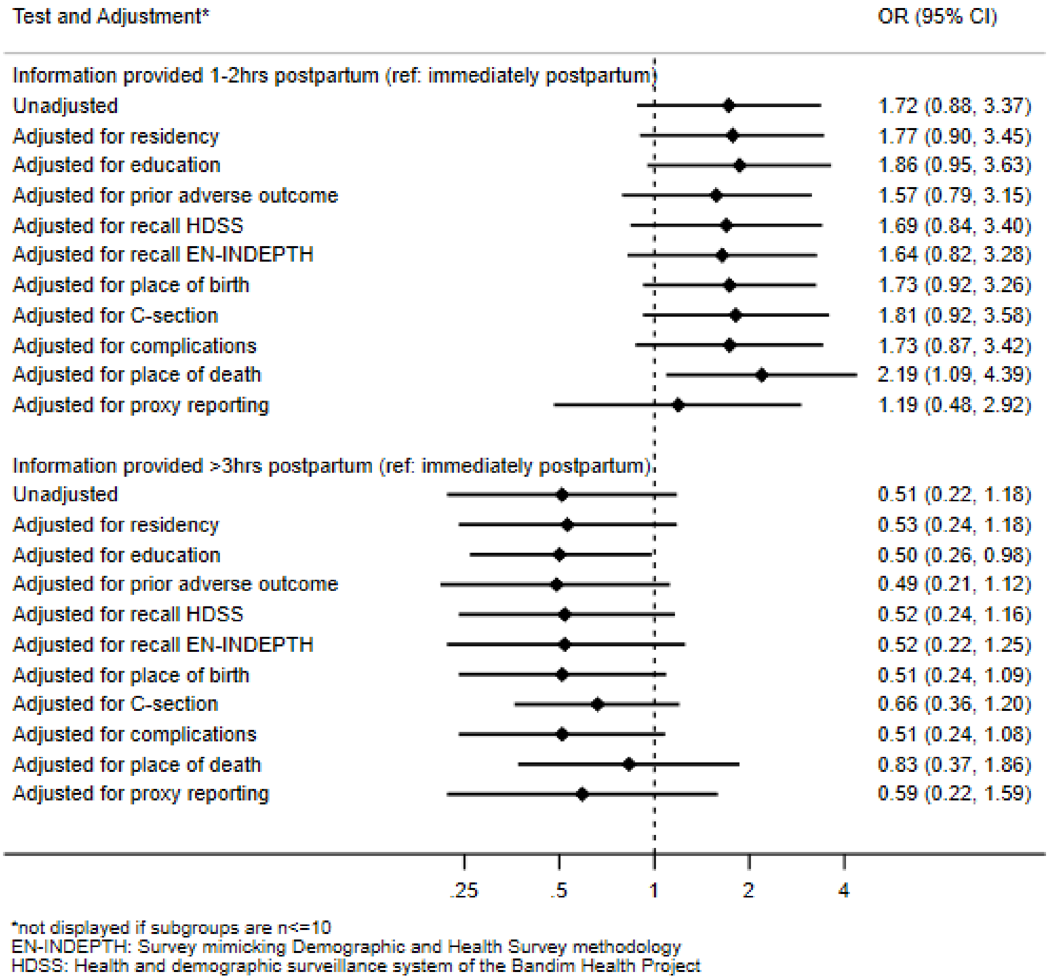

Figure S9: Multivariate regressions: Timing of information provision.

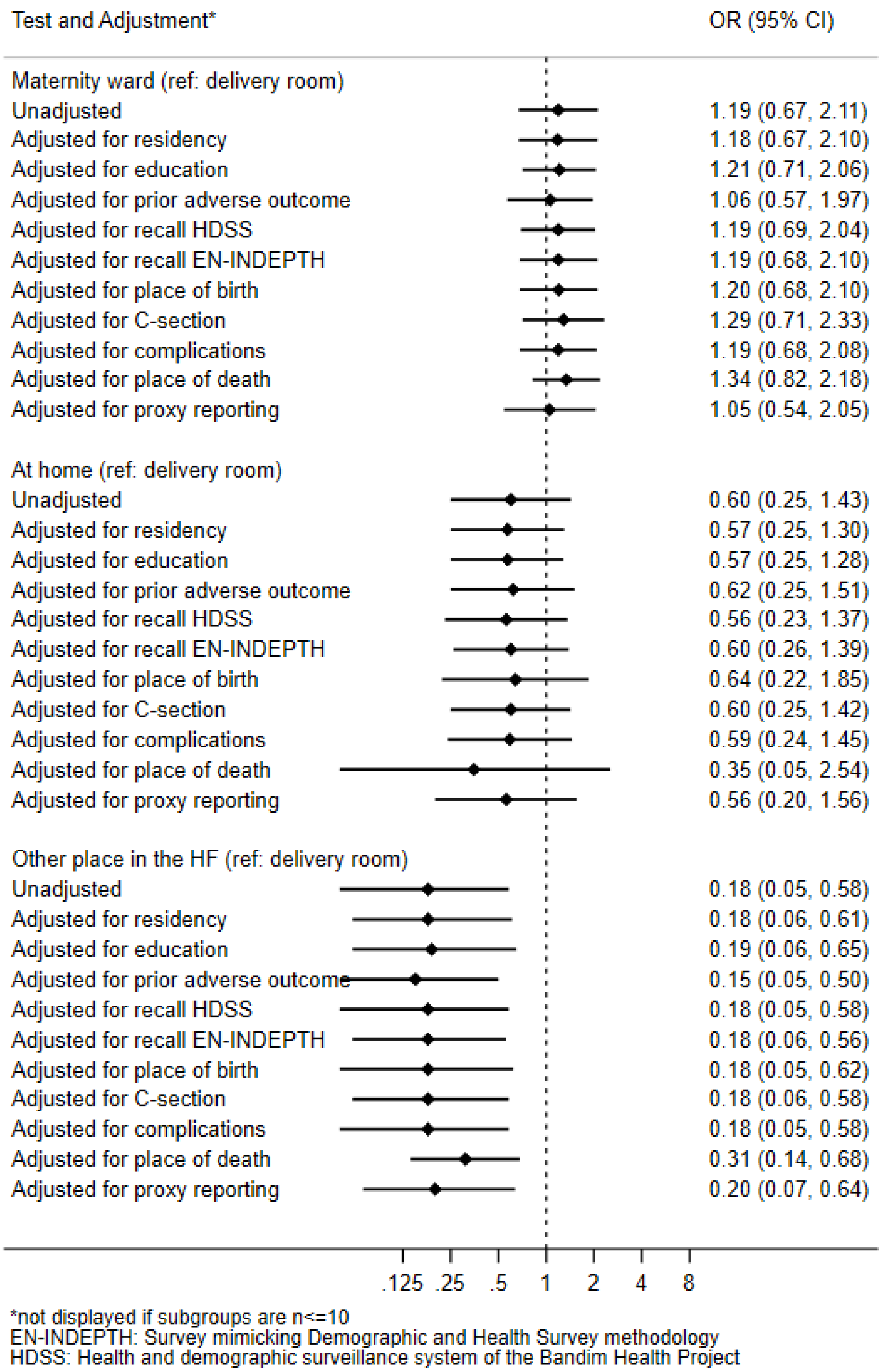

Figure S10: Multivariate regressions: Mother's location when learning about the death.

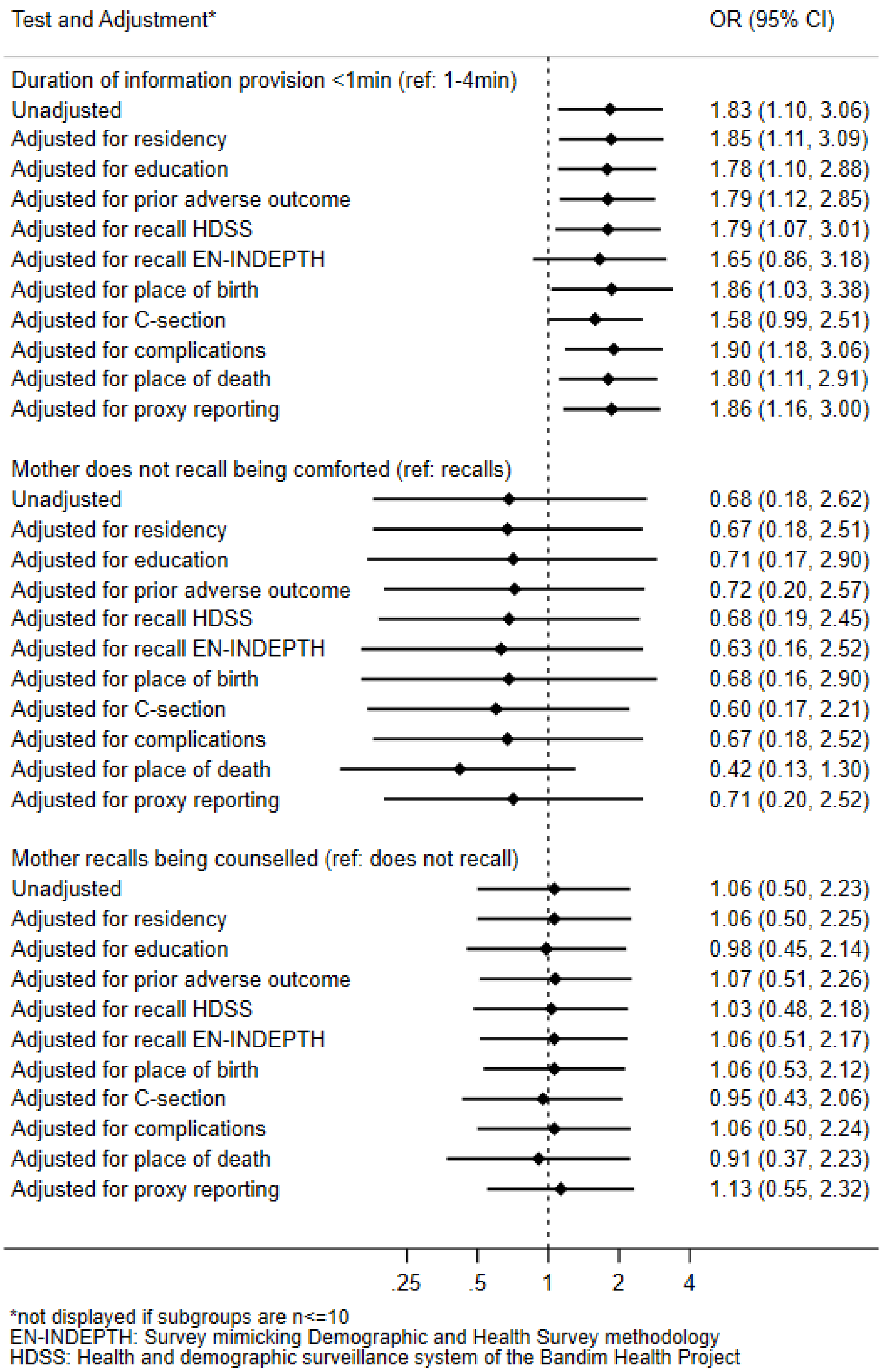

Figure S11: Multivariate regressions: Timing of information provision and provider behaviour.

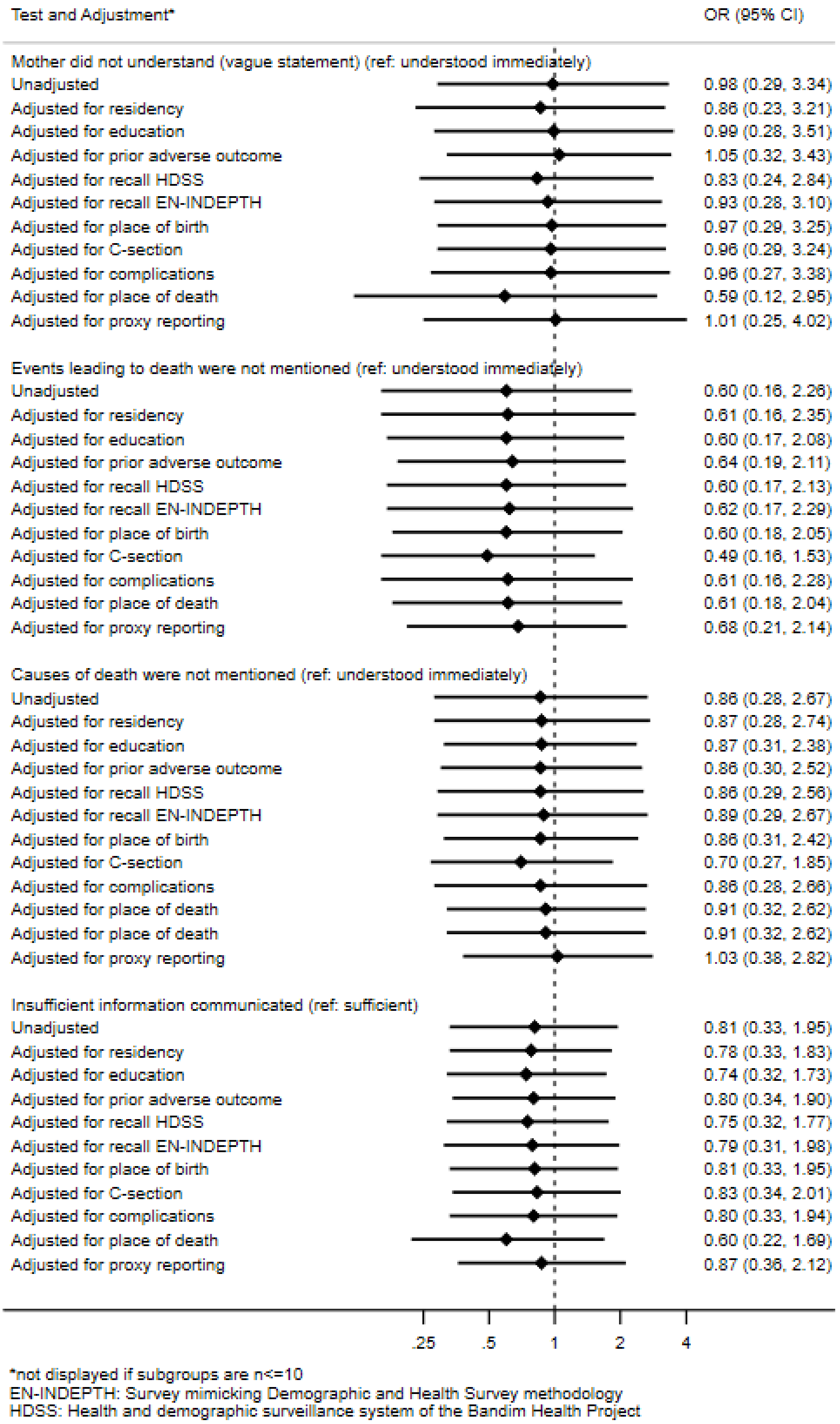

Figure S12: Multivariate regressions: Comprehensibility and completeness of information provided.

Results S5. Sensitivity analyses

Alterations of the setup of the survey commands from reporting of missing values for the standard errors to certainty, scaled and centred estimation did not change results.

3. Questionnaire S1

***General instructions**: Interviewers, please fill out blank fields in background information (Part A), read the opening script (Part B) to the respondent, and then proceed with asking her the questions listed in Part C-H. When the interview has ended, thank the respondent and summarize the interview for her by reading Part I.*

Part A: Background Information

1. Mother ID:		2. Household ID:	
3. Place of baby's birth:		4. Month and Year of baby's birth:<br>*(Verify that date of birth is on or after January 1, 2012; Otherwise, stop the survey)*	MM/YY
5. Interviewer[1]:		6. Interview Date:	
7. Start time:		8. End time:	
9. Location:			
10. Description of Setting (spaciousness, comfort, cleanliness, light level, noisiness, privacy):			

Part B: Opening Script

Thank you for agreeing to participate in our study. As previously mentioned, we are going to ask you some questions about the passing of your baby. We hope that by better understanding the communication that happens between health providers and mothers at that time, we can help health care providers learn how to give better care to mothers like you in the future. In this interview, I'd like to hear your point of view about your pregnancy and delivery experience, and how your provider communicated with you about your baby's condition.

You may experience discomfort recalling painful memories, and we can pause or stop the interview at any time. This may have happened long enough ago that may not be able to remember specific details, and that is okay, you can just tell us that you do not remember. We appreciate your honest and descriptive responses to the questions. Please let me know if anything is unclear, you can interrupt me and I can clarify for you at any time. Do you have any questions? *[pause]* We will now begin.

[1] Select one

Part C: Pregnancy Experience

Now, I would like to ask you about your pregnancy experience related to your baby who passed away.

#	Questions and probes	Coding Categories	Skip Pattern
11.	During your pregnancy, did you see anyone for antenatal care?	1. Yes<br>2. No<br>99. Don't know	 ➔ 15<br>➔ 15
12.	*(If 11=1),* Whom did you see? (*select all that apply)*	1. Doctor at facility<br>2. Midwife at facility<br>3. Nurse at facility<br>4. Facility provider (unspecified)<br>7. Traditional birth attendant<br>11. Other (specify) _____<br>99. Don't know	
13.	How many antenatal care visits did you have?	_____ # antenatal care visits<br>99. Don't know	
14.	During the pregnancy, did the antenatal care provider do any of the following for you at least once?<br>*(Read out all of the options and check yes, no, or don't know for each. Describe the procedures to help the respondent understand and recall.)*<br>14.1. Did the provider measure your blood pressure?<br>14.2. Did you give a urine sample?<br>14.3. Did you give a blood sample?<br>14.4. Did the provider tell you to eat more high energy foods and high protein foods than when not pregnant? (*Give examples of local food)*<br>14.5. Did the provider tell you about the danger signs during pregnancy?<br>14.6. Did the provider tell you where to go if you had any danger signs?	Yes No DK<br>1. □ 2.□ 99. □<br>1. □ 2.□ 99. □<br>1. □ 2.□ 99. □<br>1. □ 2.□ 99. □<br>1. □ 2.□ 99. □<br>1. □ 2.□ 99. □	
15.	Did you experience any of the following complications during your pregnancy, labor or delivery?<br>*(Read out all of the options and check yes, no, or don't know for each. Describe the procedures to help the respondent understand and recall.)*<br>15.1. Convulsions<br>15.2. High blood pressure<br>15.3 A little bleeding<br>15.4 A lot of bleeding<br>15.5. Severe lack of blood or pallor <u>and</u> shortness of breath<br>15.6. Diabetes<br>15.7. Severe headache<br>15.8. Blurred vision<br>15.9. Too weak to get out of bed<br>15.10. Severe abdominal pain (aside from labor)<br>15.11. Fast or difficult breathing<br>15.12. Puffy "Full" face<br>15.13. <u>Any</u> vaginal bleeding during labor or deliver<br>15.14. Excessive bleeding during labor or delivery<br>15.15. Fever<br>15.16. Smelly vaginal discharge	Yes No DK<br>1. □ 2.□ 99. □<br>1. □ 2.□ 99. □<br>1. □ 2.□ 99. □<br>1. □ 2.□ 99. □<br>1. □ 2.□ 99. □<br>1. □ 2.□ 99. □<br>1. □ 2.□ 99. □<br>1. □ 2.□ 99. □<br>1. □ 2.□ 99. □<br>1. □ 2.□ 99. □<br>1. □ 2.□ 99. □<br>1. □ 2.□ 99. □<br>1. □ 2.□ 99. □<br>1. □ 2.□ 99. □<br>1. □ 2.□ 99. □<br>1. □ 2.□ 99. □	

16.	How many babies did you deliver during this pregnancy?<br>*(If there was more than 1 baby, ask them to answer the remaining questions based on the baby who passed away, or the firstborn baby if both passed away)*	1. One baby<br>2. More than one baby<br>99. Don't know	
17.	Was the baby moving the last few days before birth?	1. Yes<br>2. No<br>99. Don't know	
18.	Was this your first pregnancy?	1. Yes<br>2. No<br>99. Don't know	➔ 23<br><br>➔ 23
19.	*(if 18 = 2)* Before this pregnancy, were any of your other babies stillborn?	1. Yes<br>2. No<br>99. Don't know	 ➔ 21<br>➔ 21
20.	*(If 19 = 1)* How many of your other babies were born stillborn?	______ babies<br>99. Don't know	
21.	(*If 18 = 2)* Before this pregnancy, did any of your other babies die within the first week after they were born?	1. Yes<br>2. No<br>99. Don't know	 ➔ 23<br>➔ 23
22.	*(If 21 = 1)* How many of your other babies died within the first week after they were born?	______ babies<br>99. Don't know	
23.	Was your baby born at a facility?<br>*If the birth occurred at a facility, CONTINUE TO PART D, otherwise (birth occurred at home or on the way to a facility), SKIP TO PART E*	1. Yes<br>2. No<br>99. Don't know	 ➔ Part E

Part D: Experience <u>before</u> delivery in a facility

Now, I would like to ask you about your pregnancy experience leading up to labor and delivery for your baby born on [MM/YY]

24.	Were you referred to this hospital or health facility by another health care provider? If so, who referred you?	1. Not referred<br>2. Hospital<br>3. NGO or government clinic<br>4. Private doctor/clinic<br>5. Community nurse/midwife<br>6. Community health worker<br>7. Traditional birth attendant<br>8. Religious healer<br>11. Other (specify) ______<br>99. Don't know	
25.	Did a health care provider talk to you about any possible dangers of your pregnancy related to the health of your baby before going into delivery?	1. Yes, they mentioned the baby may have minor health problems<br>2. Yes, they mentioned the baby may have serious health problems<br>3. Yes, they mentioned the baby may be stillborn<br>4. No<br>99. Don't know	 <br><br>➔ Part E<br>➔ Part E
26.	*(if 25 = 2-4*) Who discussed the dangers with you?	1. Doctor<br>2. Midwife<br>3. Nurse<br>4. Facility Provider (unspecified)	

		7. Traditional birth attendant<br>11. Other (specify) _____<br>99. Don't know	

Part E: Experience during delivery

Now, I would like to ask you about your experience during delivery

27.	How much time did the labor and delivery last? (*Read out all of the options if they're not sure)*	1. less than 6 hours<br>2. 6-11 hours<br>3. 12-18 hours<br>4. more than 18 hours<br>99. Don't know	
28.	What time of day was your baby born? (*Give examples to help describe the time i.e. "dawn" "before or after cooking")*	1. Daytime (8am-4pm)<br>2. Evening (4pm-12am)<br>3. Late night (12am-8am)<br>99. Don't know	
29.	Did you experience any of the following complications during your pregnancy, labor or delivery?<br>*(Read out all of the options and check yes, no, or don't know for each. Describe the procedures to help the respondent understand and recall.)*<br>28.1. Child delivered not head first<br>28.2. Cord delivered first<br>28.3. Cord around the child's neck<br>28.4. Any other complication (specify) __________	Yes No DK<br>1. □ 2.□ 99. □<br>1. □ 2.□ 99. □<br>1. □ 2.□ 99. □<br>1. □ 2.□ 99. □	
30.	How did you give birth?	1. Vaginally<br>2. Instrumental vaginal delivery<br>3. C-Section<br>99. Don't Know	
31.	Who delivered your baby?	1. Doctor at facility<br>2. Midwife at facility<br>3. Nurse at facility<br>4. Facility provider (unspecified)<br>7. Traditional birth attendant<br>11. Other (specify) _____<br>99. Don't know	➔ 33<br>➔ 33
32.	*(if 31 = 1-7)* How many times had you previously visited this health care provider? (*total health care visits, for any reason*)	1. None, this was the first time<br>2. 1 time<br>3. 2-4 times<br>4. 5-9 times<br>5. More than 10 times<br>99. Don't know	
33.	Were you worried about the health of your baby going into delivery?	1. No, I was not worried<br>2. Yes, I was a little worried<br>3. Yes, I was about average worried<br>4. Yes, I was very worried<br>5. Yes, I was extremely worried<br>99. Don't know	
34.	During your delivery, did this person update you about the following:	Yes No DK	

	(*Read out all of the options and check yes, no, or don't know for each. Select all that apply*)<br>33.1 Whether your baby was alive or deceased<br>33.2 Whether your baby will survive<br>33.3 Progress of your labor<br>33.4 Complications affecting your health during your labor<br>33.5 Complications affecting your baby's health during your labor	1. □ 2. □ 3. □<br>1. □ 2. □ 3. □<br>1. □ 2. □ 3. □<br>1. □ 2. □ 3. □<br><br>1. □ 2. □ 3. □	
35.	Did you have any questions about the progress of your labor during your delivery?	1. Yes<br>2. No<br>99. Don't Know	
36.	Did you ask this person any questions about the progress of you labor during your delivery?	1. Yes, I asked questions<br>2. No, I was not able to<br>3. No, I did not have questions<br>99. Don't Know	
37.	Did you have any questions about how your baby was doing during your delivery?	1. Yes<br>2. No<br>99. Don't Know	
38.	Did you ask this person any questions about how your baby was doing during your delivery?	1. Yes, I asked questions<br>2. No, I was not able to<br>3. No, I did not have questions<br>99. Don't Know	

Part F: Experience <u>after</u> delivery

Now, I would like to ask you about your experience after delivery.

39.	After the delivery, was your baby close enough to you for you to see, hear, or feel the baby?	1. Yes<br>2. No<br>99. Don't Know	 ➔ 43<br>➔ 43
40.[2]	*(If 39 = 1)* Immediately after you gave birth, did you personally see, hear, or feel your baby showing any of the following signs of life?<br>*(Read out all of the options and check yes, no, or don't know for each. If yes, ask whether they saw, heard, or felt the sign of life. Select all that apply)*<br>40.1 Baby was crying<br>40.2 Baby was moving<br>40.3 Baby was breathing<br>40.4 Baby had heartbeat<br><br>*If any of 40 = 1, continue to question 41; otherwise, skip to question 43.*	<u>Yes No DK</u><br>1. □ 2.□ 99. □<br>1. □ 2.□ 99. □<br>1. □ 2.□ 99. □<br>1. □ 2.□ 99. □	➔ 43 if all = 2 or 99
41.	(*If any of 40 = yes*) Were there any bruises or signs of injury on the baby's body at birth?	1. Yes<br>2. No<br>99. Don't Know	
42.	(*If any of 40 = yes*) Was the baby's skin peeling or showing signs of decay?	1. Yes<br>2. No<br>99. Don't Know	

[2] 40-42 Interviewer manually skips

43.	*(If 39=2 or 99, OR all of 40 = no)* You mentioned that you did not feel your baby. Can you tell me the main reason you did not hold your baby after birth?	1. I didn't want to<br>2. The health care provider did not allow me to<br>3. Family member did not allow me to<br>4. I could not for cultural or religious reasons<br>11. Other (specify) ______<br>99. Don't Know	
44.[3]	*(Ask the first part of the question, and check "No" if the mother was not told the following. If she was told, ask the second part of the question and check "yes (affirmative)" if told the baby DID show sign, yes (negative) if told the baby DID NOT show sign)*<br>43.1 Were you told whether or not the baby was crying? [If yes] Did your baby cry?<br>43.2 Were you told whether or not the baby was moving? [If yes] Did your baby move?<br>43.3 Were you told whether or not the baby was breathing? [If yes] Did your baby breath?<br>43.4 Were you told whether or not the baby had a heartbeat? [If yes] Did your baby have a heartbeat?<br>*If any of 44 = 1 or 2, continue to question 45; otherwise, skip to Part G.*	Yes Yes No DK (affirmative) (negative)<br>1. □ 2.□ 3.□ 99. □<br>1. □ 2.□ 3.□ 99. □<br>1. □ 2.□ 3.□ 99. □<br>1. □ 2.□ 3.□ 99. □	➔ Part G if all = 3 or 99
45.	*If any part of 44 ="1 or 2": Who told you this information? (select all that apply)*	1. Doctor at facility<br>2. Midwife at facility<br>3. Nurse at facility<br>4. Facility provider (unspecified)<br>5. Traditional birth attendant<br>11. Other (specify) _____<br>99. Don't know	

Part G: Communication of vital status

Now, I would like to ask you about your experience when you learned about the death of your baby. I understand this may be difficult for you, and you may feel uncomfortable recalling painful memories. We can pause or stop the interview at any time.

46.	When did your baby pass?	1. Before birth<br>2. Immediately after birth<br>3. Within one week after birth<br>4. More than one week after birth<br>99. Don't Know	
47.	Where did your baby pass?	1. At the hospital of delivery<br>2. Other health facility<br>3. At home<br>4. On the way to a health facility/hospital<br>11. Other (specify) _____<br>99. Don't Know	
48.	Where were you when you were told your baby had passed?	1. Delivery room<br>2. Post-delivery recovery room<br>3. Maternity ward	

[3] 44 Interviewer manually skips

		4. Home<br>11. Other place in facility (specify) ______<br>12. Other place outside facility (specify) ____________<br>99. Don't Know	
49.	Who else was in the room when you were told your baby had passed? (*Select all that apply)*	1. I was alone<br>2. My family<br>3. Other people I did not know<br>99. Don't Know	
50.	Who was the first person to tell you that your baby had died? *(Confirm the type of provider by asking the mother additional information (ex: what were the color of their scrubs, where in the ward did she see them.)*<br>*If 50 = 1-7, continue to question 51; otherwise, skip to Part H.*	1. Doctor at facility<br>2. Midwife at facility<br>3. Nurse at facility<br>4. Facility provider (unspecified)<br>7. Traditional birth attendant<br>11. Other (specify) ______<br>99. Don't know	➔ Part H<br>➔ Part H
51.	How many times had you previously visited this person? (*total health care visits, for any reason*)	1. None, this was the first time<br>2. 1 time<br>3. 2-4 times<br>4. 5-9 times<br>5. More than 10 times<br>99. Don't know	
52.	What language did this person speak?	1. Kriol<br>2. Portuguese<br>11. Other (specify) _______<br>99. Don't Know	
53.	How long did this person spend talking to you about your baby's passing?	1. Less than 1 minute<br>2. 1-4 minutes<br>3. 5-15 minutes<br>4. More than 15 minutes<br>99. Don't Know	
54.	How soon after delivery did this conversation about your baby's death occur?	1. Immediately after delivery<br>2. 1-2 hours after delivery<br>3. 3-24 hours after delivery<br>4. More than 24 hours after delivery<br>5. They didn't tell me anything<br>99. Don't Know	
55.	Did this person talk with you about the death of your baby in a way that you could understand?	1. No, I overheard them talking to another person<br>2. No, they said a vague statement that might have suggested my baby died<br>3. No, they used medical words I did not understand<br>4. Yes, but I had to ask for more information<br>5. Yes, I understood them immediately<br>99. Don't Know	

56.	Did this person communicate the events that led to the death in a way you could understand?	1. No, they did not mention the events leading up to death<br>2. No, they were uncertain of the events leading up to death<br>3. No, they used medical words I did not understand<br>4. Yes, but only after I asked for more information<br>5. Yes, I understood the events leading up to death immediately<br>99. Don't Know	
57.	Did this person communicate the cause of death to you in a way that you could understand?	1. No, they did not mention the cause of death<br>2. No, they were uncertain of the cause of death<br>3. No, they used medical words I did not understand<br>4. Yes, but only after I asked for more information<br>5. Yes, I understood the cause of death immediately<br>99. Don't Know	
58.	Did this person tell you all the information you wanted to know?<br><br>*Probe: Would you have liked to know more?*	1. Yes, they told me what I needed to know<br>2. No, they told me too much information<br>3. No, they did not tell me enough information<br>99. Don't know	
59.	How did the person who told you your baby had died behave after they gave you the news? *(Read out all the options by groups (1-3, 4-5, 6-7, 99), and select all that apply)*	1. The tried to comfort me<br>2. They acknowledged my suffering<br>3. They stayed, but did not counsel me<br>4. They said hurtful comments<br>5. They blamed me for the death<br>6. They tried to hide or suppress my grief<br>7. They left immediately without saying or doing anything more<br>99. Don't know	
60.	Besides concentrating on you, was this person doing other things while telling you about the death of your baby?	1. No, they paid full attention to me<br>2. Yes, they were distracted by ______<br>99. Don't know	
61.	Did you worry that this person may share information about your baby's death with other people?	1. Yes, I was worried<br>2. No, I was not worried<br>99. Don't know	

Part H: Culture and taboo

Thank you for sharing your personal delivery experience. Now, I would like to ask about your experience back in your community after the loss of your baby. I would like you to think about your community, and the beliefs people have around babies who die late in pregnancy or around the time of birth.

62.	Did you grieve for your child? *(select all that apply)*<br>*If 62 = 2-3, complete the following question 63; otherwise, skip to 64.*	1. No, I did not grieve<br>2. Yes, I grieved alone<br>3. Yes, I grieved with others<br>99. Don't know	➔ 64<br><br><br>➔ 64
63.	Who did you grieve with?	1. Spouse/partner<br>2. Other family<br>3. Friends<br>4. Community<br>5. Don't know	
64.	What happened to the baby's body?	1. It was left at the health facility<br>2. It was buried<br>3. It was thrown away<br>11. Other (specify): _______<br>99. Don't know	
65.	Was there a funeral for the baby?	1. Yes<br>2. No<br>99. Don't know	
66.	What do people in your community believe or say about women who have babies who die late in pregnancy or around the time of birth? Tell me about what usually happens. I would also like to hear about your personal experience after your baby died. *(open-ended, record key quotes verbatim)*<br><br>*Probe: How do people in your community treat these women?*<br><br>*Probe: What advice do people in your community give these women?*		

Part I: Conclusion

Thank you very much for taking the time and share your experience with us. It is a privilege to be able to speak with you. Please let me know if you have any questions or concerns, or you can also contact our local Principal Investigator (Professor Ane Fisker, [number]). Thank you again for your time and important contributions to this study.

4. References

1 Baschieri A, Gordeev VS, Akuze J, Kwesiga D, Blencowe H, Cousens S, et al. "Every Newborn-INDEPTH" (EN-INDEPTH) study protocol for a randomised comparison of household survey modules for measuring stillbirths and neonatal deaths in five Health and Demographic Surveillance sites. J Glob Health. 2019;9:010901.

2 Akuze J, Blencowe H, Waiswa P, Baschieri A, Gordeev VS, Kwesiga D, et al. Randomised comparison of two household survey modules for measuring stillbirths and neonatal deaths in five countries: the Every Newborn-INDEPTH study. Lancet Glob Health. 2020;8:e555-e66.

3 Nareeba T, Dzabeng F, Alam N, Biks GA, Thysen SM, Akuze J, et al. Neonatal and child mortality data in retrospective population-based surveys compared with prospective demographic surveillance: EN-INDEPTH study. Population Health Metrics. 2021;19:7.

4 Jensen AM. Measuring early neonatal mortality in low-income countries. Bandim Health Project, Research Unit OPEN, Department of Clinical Research: University of Southern Denmark; 2023.

5 Thysen SM, Fernandes M, Benn CS, Aaby P, Fisker AB. Cohort profile: Bandim Health Project's (BHP) rural Health and Demographic Surveillance System (HDSS) - a nationally representative HDSS in Guinea-Bissau. BMJ Open. 2019;9:e028775.

6 Bjerregaard-Andersen M, Lund N, Joergensen ASP, Jepsen FS, Unger HW, Mane M, et al. Stillbirths in urban Guinea-Bissau: A hospital- and community-based study. PLoS One. 2018;13:e0197680.
